# Integrating large-scale meta-GWAS and PigGTEx resources to decipher the genetic basis of 232 complex traits in pigs

**DOI:** 10.1093/nsr/nwaf048

**Published:** 2025-02-17

**Authors:** Zhiting Xu, Qing Lin, Xiaodian Cai, Zhanming Zhong, Jinyan Teng, Bingjie Li, Haonan Zeng, Yahui Gao, Zexi Cai, Xiaoqing Wang, Liangyu Shi, Xue Wang, Yi Wang, Zipeng Zhang, Yu Lin, Shuli Liu, Hongwei Yin, Zhonghao Bai, Chen Wei, Jun Zhou, Wenjing Zhang, Xiaoke Zhang, Shaolei Shi, Jun Wu, Shuqi Diao, Yuqiang Liu, Xiangchun Pan, Xueyan Feng, Ruiqi Liu, Zhanqin Su, Chengjie Chang, Qianghui Zhu, Yuwei Wu, The PigGTEx Consortium, Zhongyin Zhou, Lijing Bai, Kui Li, Qishan Wang, Yuchun Pan, Zhong Xu, Xianwen Peng, Shuqi Mei, Delin Mo, Xiaohong Liu, Hao Zhang, Xiaolong Yuan, Yang Liu, George E Liu, Guosheng Su, Goutam Sahana, Mogens Sandø Lund, Li Ma, Ruidong Xiang, Xia Shen, Pinghua Li, Ruihua Huang, Maria Ballester, Daniel Crespo-Piazuelo, Marcel Amills, Alex Clop, Peter Karlskov-Mortensen, Merete Fredholm, Guoqing Tang, Mingzhou Li, Xuewei Li, Xiangdong Ding, Jiaqi Li, Yaosheng Chen, Qin Zhang, Yunxiang Zhao, Fuping Zhao, Lingzhao Fang, Zhe Zhang

**Affiliations:** State Key Laboratory of Swine and Poultry Breeding Industry, National Engineering Research Center for Breeding Swine Industry, Guangdong Provincial Key Lab of Agro-Animal Genomics and Molecular Breeding, College of Animal Science, South China Agricultural University, Guangzhou 510642, China; State Key Laboratory of Swine and Poultry Breeding Industry, National Engineering Research Center for Breeding Swine Industry, Guangdong Provincial Key Lab of Agro-Animal Genomics and Molecular Breeding, College of Animal Science, South China Agricultural University, Guangzhou 510642, China; State Key Laboratory of Swine and Poultry Breeding Industry, National Engineering Research Center for Breeding Swine Industry, Guangdong Provincial Key Lab of Agro-Animal Genomics and Molecular Breeding, College of Animal Science, South China Agricultural University, Guangzhou 510642, China; State Key Laboratory of Swine and Poultry Breeding Industry, National Engineering Research Center for Breeding Swine Industry, Guangdong Provincial Key Lab of Agro-Animal Genomics and Molecular Breeding, College of Animal Science, South China Agricultural University, Guangzhou 510642, China; State Key Laboratory of Swine and Poultry Breeding Industry, National Engineering Research Center for Breeding Swine Industry, Guangdong Provincial Key Lab of Agro-Animal Genomics and Molecular Breeding, College of Animal Science, South China Agricultural University, Guangzhou 510642, China; Department of Animal and Veterinary Sciences, The Roslin Institute Building, Scotland's Rural College (SRUC), Easter Bush, Midlothian EH25 9RG, UK; State Key Laboratory of Swine and Poultry Breeding Industry, National Engineering Research Center for Breeding Swine Industry, Guangdong Provincial Key Lab of Agro-Animal Genomics and Molecular Breeding, College of Animal Science, South China Agricultural University, Guangzhou 510642, China; State Key Laboratory of Swine and Poultry Breeding Industry, National Engineering Research Center for Breeding Swine Industry, Guangdong Provincial Key Lab of Agro-Animal Genomics and Molecular Breeding, College of Animal Science, South China Agricultural University, Guangzhou 510642, China; Animal Genomics and Improvement Laboratory, Henry A. Wallace Beltsville Agricultural Research Center, Agricultural Research Service (ARS), U.S.Department of Agriculture (USDA), Beltsville, Maryland 20705, USA; Department of Animal and Avian Sciences, University of Maryland, College Park, Maryland 20742, USA; Center for Quantitative Genetics and Genomics (QGG), Aarhus University, Aarhus 8000, Denmark; Institute of Animal Science, Chinese Academy of Agricultural Sciences, Beijing 100193, China; Institute of Animal Science, Chinese Academy of Agricultural Sciences, Beijing 100193, China; College of Animal Science and Technology, China Agricultural University, Beijing 100193, China; College of Animal Science and Technology, China Agricultural University, Beijing 100193, China; College of Animal Science and Technology, China Agricultural University, Beijing 100193, China; Key Laboratory of Agricultural Bioinformatics, Ministry of Education, College of Animal Science and Technology, Sichuan Agricultural University, Chengdu 611130, China; Westlake Laboratory of Life Sciences and Biomedicine, Hangzhou 310024, China; Shenzhen Branch, Guangdong Laboratory for Lingnan Modern Agriculture, Genome Analysis Laboratory of the Ministry of Agriculture, Agricultural Genomics Institute at Shenzhen, Chinese Academy of Agricultural Sciences, Shenzhen 518124, China; Center for Quantitative Genetics and Genomics (QGG), Aarhus University, Aarhus 8000, Denmark; State Key Laboratory of Swine and Poultry Breeding Industry, National Engineering Research Center for Breeding Swine Industry, Guangdong Provincial Key Lab of Agro-Animal Genomics and Molecular Breeding, College of Animal Science, South China Agricultural University, Guangzhou 510642, China; State Key Laboratory of Swine and Poultry Breeding Industry, National Engineering Research Center for Breeding Swine Industry, Guangdong Provincial Key Lab of Agro-Animal Genomics and Molecular Breeding, College of Animal Science, South China Agricultural University, Guangzhou 510642, China; State Key Laboratory of Swine and Poultry Breeding Industry, National Engineering Research Center for Breeding Swine Industry, Guangdong Provincial Key Lab of Agro-Animal Genomics and Molecular Breeding, College of Animal Science, South China Agricultural University, Guangzhou 510642, China; State Key Laboratory of Swine and Poultry Breeding Industry, National Engineering Research Center for Breeding Swine Industry, Guangdong Provincial Key Lab of Agro-Animal Genomics and Molecular Breeding, College of Animal Science, South China Agricultural University, Guangzhou 510642, China; State Key Laboratory of Swine and Poultry Breeding Industry, National Engineering Research Center for Breeding Swine Industry, Guangdong Provincial Key Lab of Agro-Animal Genomics and Molecular Breeding, College of Animal Science, South China Agricultural University, Guangzhou 510642, China; State Key Laboratory of Swine and Poultry Breeding Industry, National Engineering Research Center for Breeding Swine Industry, Guangdong Provincial Key Lab of Agro-Animal Genomics and Molecular Breeding, College of Animal Science, South China Agricultural University, Guangzhou 510642, China; State Key Laboratory of Swine and Poultry Breeding Industry, National Engineering Research Center for Breeding Swine Industry, Guangdong Provincial Key Lab of Agro-Animal Genomics and Molecular Breeding, College of Animal Science, South China Agricultural University, Guangzhou 510642, China; State Key Laboratory of Swine and Poultry Breeding Industry, National Engineering Research Center for Breeding Swine Industry, Guangdong Provincial Key Lab of Agro-Animal Genomics and Molecular Breeding, College of Animal Science, South China Agricultural University, Guangzhou 510642, China; State Key Laboratory of Swine and Poultry Breeding Industry, National Engineering Research Center for Breeding Swine Industry, Guangdong Provincial Key Lab of Agro-Animal Genomics and Molecular Breeding, College of Animal Science, South China Agricultural University, Guangzhou 510642, China; State Key Laboratory of Swine and Poultry Breeding Industry, National Engineering Research Center for Breeding Swine Industry, Guangdong Provincial Key Lab of Agro-Animal Genomics and Molecular Breeding, College of Animal Science, South China Agricultural University, Guangzhou 510642, China; State Key Laboratory of Swine and Poultry Breeding Industry, National Engineering Research Center for Breeding Swine Industry, Guangdong Provincial Key Lab of Agro-Animal Genomics and Molecular Breeding, College of Animal Science, South China Agricultural University, Guangzhou 510642, China; State Key Laboratory of Swine and Poultry Breeding Industry, National Engineering Research Center for Breeding Swine Industry, Guangdong Provincial Key Lab of Agro-Animal Genomics and Molecular Breeding, College of Animal Science, South China Agricultural University, Guangzhou 510642, China; State Key Laboratory of Swine and Poultry Breeding Industry, National Engineering Research Center for Breeding Swine Industry, Guangdong Provincial Key Lab of Agro-Animal Genomics and Molecular Breeding, College of Animal Science, South China Agricultural University, Guangzhou 510642, China; State Key Laboratory of Swine and Poultry Breeding Industry, National Engineering Research Center for Breeding Swine Industry, Guangdong Provincial Key Lab of Agro-Animal Genomics and Molecular Breeding, College of Animal Science, South China Agricultural University, Guangzhou 510642, China; State Key Laboratory of Swine and Poultry Breeding Industry, National Engineering Research Center for Breeding Swine Industry, Guangdong Provincial Key Lab of Agro-Animal Genomics and Molecular Breeding, College of Animal Science, South China Agricultural University, Guangzhou 510642, China; State Key Laboratory of Genetic Resources and Evolution, Kunming Institute of Zoology, Chinese Academy of Sciences, Kunming 650223, China; Shenzhen Branch, Guangdong Laboratory for Lingnan Modern Agriculture, Genome Analysis Laboratory of the Ministry of Agriculture, Agricultural Genomics Institute at Shenzhen, Chinese Academy of Agricultural Sciences, Shenzhen 518124, China; Shenzhen Branch, Guangdong Laboratory for Lingnan Modern Agriculture, Genome Analysis Laboratory of the Ministry of Agriculture, Agricultural Genomics Institute at Shenzhen, Chinese Academy of Agricultural Sciences, Shenzhen 518124, China; Department of Animal Science, College of Animal Sciences, Zhejiang University, Hangzhou 310058, China; Department of Animal Science, College of Animal Sciences, Zhejiang University, Hangzhou 310058, China; Hubei Key Laboratory of Animal Embryo and Molecular Breeding, Institute of Animal Husbandry and Veterinary, Hubei Provincial Academy of Agricultural Sciences, Wuhan 430064, China; Hubei Key Laboratory of Animal Embryo and Molecular Breeding, Institute of Animal Husbandry and Veterinary, Hubei Provincial Academy of Agricultural Sciences, Wuhan 430064, China; Hubei Key Laboratory of Animal Embryo and Molecular Breeding, Institute of Animal Husbandry and Veterinary, Hubei Provincial Academy of Agricultural Sciences, Wuhan 430064, China; State Key Laboratory of Biocontrol, School of Life Sciences, Sun Yat-sen University, Guangzhou 510275, China; State Key Laboratory of Biocontrol, School of Life Sciences, Sun Yat-sen University, Guangzhou 510275, China; State Key Laboratory of Swine and Poultry Breeding Industry, National Engineering Research Center for Breeding Swine Industry, Guangdong Provincial Key Lab of Agro-Animal Genomics and Molecular Breeding, College of Animal Science, South China Agricultural University, Guangzhou 510642, China; State Key Laboratory of Swine and Poultry Breeding Industry, National Engineering Research Center for Breeding Swine Industry, Guangdong Provincial Key Lab of Agro-Animal Genomics and Molecular Breeding, College of Animal Science, South China Agricultural University, Guangzhou 510642, China; College of Animal Science and Technology, Nanjing Agricultural University, Nanjing 210095, China; Animal Genomics and Improvement Laboratory, Henry A. Wallace Beltsville Agricultural Research Center, Agricultural Research Service (ARS), U.S.Department of Agriculture (USDA), Beltsville, Maryland 20705, USA; Center for Quantitative Genetics and Genomics (QGG), Aarhus University, Aarhus 8000, Denmark; Center for Quantitative Genetics and Genomics (QGG), Aarhus University, Aarhus 8000, Denmark; Center for Quantitative Genetics and Genomics (QGG), Aarhus University, Aarhus 8000, Denmark; Department of Animal and Avian Sciences, University of Maryland, College Park, Maryland 20742, USA; Faculty of Veterinary & Agricultural Science, University of Melbourne, Parkville, VIC 3010, Australia; Agriculture Victoria Research, AgriBio Centre for AgriBiosciences, Bundoora, VIC 3083, Australia; State Key Laboratory of Genetic Engineering, School of Life Sciences, Fudan University, Shanghai 200438, China; Center for Intelligent Medicine Research, Greater Bay Area Institute of Precision Medicine (Guangzhou), Fudan University, Guangzhou 510000, China; Centre for Global Health Research, Usher Institute, University of Edinburgh, Edinburgh EH16 4UX, UK; Institute of Swine Science, Nanjing Agricultural University, Nanjing 210095, China; Key Laboratory in Nanjing for Evaluation and Utilization of Livestock and Poultry (Pigs) Resources, Ministry of Agriculture and Rural Areas, Nanjing 210095, China; Institute of Swine Science, Nanjing Agricultural University, Nanjing 210095, China; Key Laboratory in Nanjing for Evaluation and Utilization of Livestock and Poultry (Pigs) Resources, Ministry of Agriculture and Rural Areas, Nanjing 210095, China; Animal Breeding and Genetics Programme, Institut de Recerca i Tecnologia Agroalimentàries (IRTA), Torre Marimon, Caldes de Montbui 08140, Spain; Animal Breeding and Genetics Programme, Institut de Recerca i Tecnologia Agroalimentàries (IRTA), Torre Marimon, Caldes de Montbui 08140, Spain; Department of Animal Genetics, Centre for Research in Agricultural Genomics (CRAG), CSIC-IRTA-UAB-UB, Campus de la Universitat Autònoma de Barcelona, Bellaterra 08193, Spain; Departament de Ciència Animal i dels Aliments, Universitat Autònoma de Barcelona, Bellaterra 08193, Spain; Department of Animal Genetics, Centre for Research in Agricultural Genomics (CRAG), CSIC-IRTA-UAB-UB, Campus de la Universitat Autònoma de Barcelona, Bellaterra 08193, Spain; Animal Genetics and Breeding, Department of Veterinary and Animal Sciences, University of Copenhagen, Frederiksberg C 1870, Denmark; Animal Genetics and Breeding, Department of Veterinary and Animal Sciences, University of Copenhagen, Frederiksberg C 1870, Denmark; Key Laboratory of Agricultural Bioinformatics, Ministry of Education, College of Animal Science and Technology, Sichuan Agricultural University, Chengdu 611130, China; Key Laboratory of Agricultural Bioinformatics, Ministry of Education, College of Animal Science and Technology, Sichuan Agricultural University, Chengdu 611130, China; Key Laboratory of Agricultural Bioinformatics, Ministry of Education, College of Animal Science and Technology, Sichuan Agricultural University, Chengdu 611130, China; College of Animal Science and Technology, China Agricultural University, Beijing 100193, China; State Key Laboratory of Swine and Poultry Breeding Industry, National Engineering Research Center for Breeding Swine Industry, Guangdong Provincial Key Lab of Agro-Animal Genomics and Molecular Breeding, College of Animal Science, South China Agricultural University, Guangzhou 510642, China; State Key Laboratory of Biocontrol, School of Life Sciences, Sun Yat-sen University, Guangzhou 510275, China; College of Animal Science and Technology, Shandong Agricultural University, Tai'an 271018, China; College of Animal Science and Technology, Guangxi University, Nanning 530004, China; Institute of Animal Science, Chinese Academy of Agricultural Sciences, Beijing 100193, China; Center for Quantitative Genetics and Genomics (QGG), Aarhus University, Aarhus 8000, Denmark; State Key Laboratory of Swine and Poultry Breeding Industry, National Engineering Research Center for Breeding Swine Industry, Guangdong Provincial Key Lab of Agro-Animal Genomics and Molecular Breeding, College of Animal Science, South China Agricultural University, Guangzhou 510642, China

**Keywords:** pig, meta-GWAS, PigGTEx, molQTL, complex traits

## Abstract

Understanding the molecular and cellular mechanisms underlying complex traits in pigs is crucial for enhancing genetic gain via artificial selection and utilizing pigs as models for human disease and biology. Here, we conducted comprehensive genome-wide association studies (GWAS) followed by a cross-breed meta-analysis for 232 complex traits and a within-breed meta-analysis for 12 traits, using 28.3 million imputed sequence variants in 70 328 animals across 14 pig breeds. We identified 6878 quantitative trait loci (QTL) for 139 complex traits. Leveraging the Pig Genotype-Tissue Expression resource, we systematically investigated the biological context and regulatory mechanisms behind these trait-QTLs, ultimately prioritizing 14 829 variant-gene-tissue-trait regulatory circuits. For instance, rs344053754 regulates *UGT2B31* expression in the liver and intestines, potentially by modulating enhancer activity, ultimately influencing litter weight at weaning in pigs. Furthermore, we observed conservation of certain genetic and regulatory mechanisms underlying complex traits between humans and pigs. Overall, our cross-breed meta-GWAS in pigs provides invaluable resources and novel insights into the genetic regulatory and evolutionary mechanisms of complex traits in mammals.

## INTRODUCTION

Pigs are one of the most important sources of animal protein worldwide, with an global pork production reached 124.5 million tons in 2023 [1]. In the past decade, genomic selection has been extensively employed to improve pig breeding [2]. Recently, genome editing has been increasingly applied to protect pigs against porcine reproductive and respiratory syndrome virus [3] and transmissible gastroenteritis virus [4]. A deeper comprehension of the genetic underpinnings of complex traits in pigs will further enable us to genetically maximize their production efficiency [5] and improve their health and welfare [6,7], while minimizing environmental impacts [8,9] through developing more advanced precision breeding techniques. Besides serving as a primary source of animal protein for humans [10], pigs are widely accepted as a valuable model for studying human biology and diseases, including Alzheimer's disease [11], cardiovascular disease [12], wound healing [13,14], human reproduction [15], human gastrointestinal tract [16], dry eye [17] and immunological studies [18–20]. Therefore, investigating the genetic and biological architecture of complex traits and diseases in pigs is crucial for both agricultural advancements and biomedical research.

A genome-wide association study (GWAS) is a prevalent approach for dissecting the genetic basis of complex traits and diseases [21–23]. As of 28 April 2024, the Pig quantitative trait loci (QTL) database (QTLdb) has gathered 48 875 QTL for 673 distinct traits [24]. However, the causal variants and genes underlying most of these QTL regions remain unknown due to extensive linkage disequilibrium (LD) among genetic variants and the lack of functional annotations [25]. Cross-ancestry meta-analysis of GWAS (meta-GWAS) has emerged as an effective strategy to uncover trait-associated variants shared across populations and accelerate statistical fine mapping of causal variants and genes through reducing LD [23,26–28]. In addition, the majority of genetic variants identified in GWAS are located in non-coding regions [29] and are notably enriched in *cis*-regulatory elements such as promoters and enhancers [30,31], as well as in gene expression QTL (eQTL) within relevant tissues [32]. Therefore, it is of great interest to prioritize causal variants, genes, pathways and tissues of complex traits in livestock through systematically integrating GWAS results with relevant functional annotation data, including those from the Functional Annotation of ANimal Genomes (FAANG) project [33] and the Farm Animal Genotype-Tissue Expression (FarmGTEx) project [34].

**Figure 1.**
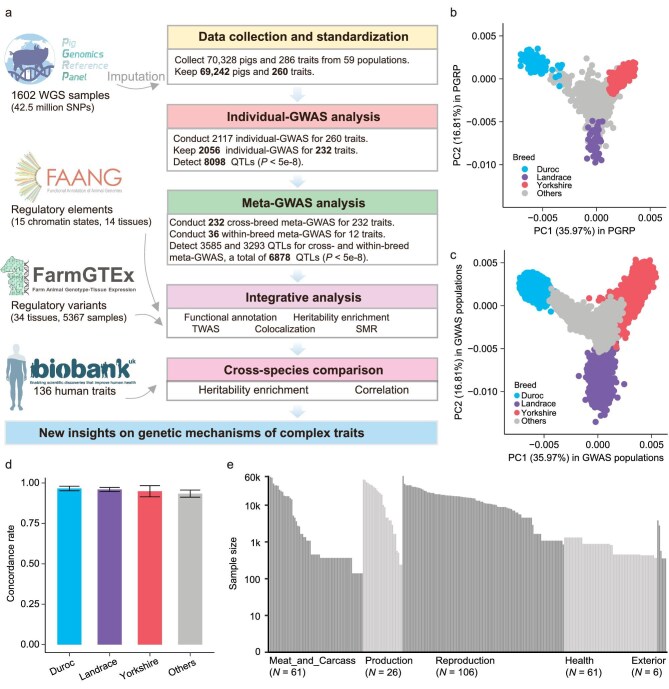
Overall study design and summary of genotypes and phenotypes. (a) Overview of study design. WGS: whole genome sequence. GWAS: genome-wide association study. TWAS: transcriptome-wide association study. SMR: summary data-based Mendelian randomization. (b and c) Principal component (PC) analysis of Pig Genomics Reference Panel (PGRP) (b) and GWAS populations (c), which were conducted based on 57 600 individuals (samples with raw genotype data) and a total of 1603 shared array SNPs using PLINK (v1.90) [84]. The first two PCs were plotted using the *geom_point* function from the ggplot2 (v3.3.6) package in R (v4.1.2). (d) The imputation accuracy (concordance rate between the imputed and observed genotypes) of PGRP in GWAS data. This was evaluated on chromosome 6 with 20 times 5-fold cross-validation using Chip array data from 60 720 pigs in 53 GWAS populations that had individual-level genotype data. Error bars indicate the standard deviation. (e) The total sample size was collected for each trait. Traits were classified into five main trait-categories.

Here, we collected and analyzed phenotypes and genotypes from 70 328 pigs across 59 populations, representing 14 distinct pig breeds, to identify genetic variants underlying various complex traits in pigs. After imputing genotypes to the sequence level using a multi-breed genotype imputation reference panel [34], we conducted a comprehensive cross-breed meta-GWAS for 232 complex traits and a within-breed meta-GWAS for 12 traits. Subsequently, we integrated regulatory elements from the FAANG project [33] and molecular QTL (molQTL) from the Pig Genotype-Tissue Expression (PigGTEx) project [34] to systematically elucidate the molecular and cellular mechanisms underlying complex traits in pigs. To explore the potential of pigs as models for human complex traits and diseases, we compared the genetic regulation of 136 human complex phenotypes with our 126 pig meta-GWAS, focusing on those with significant GWAS loci and sample sizes greater than 1000. All the resources are freely available in the PigBiobank web portal (https://pigbiobank.farmgtex.org) (Fig. 1a).

## RESULTS

### Summary of genotypes and phenotypes

After excluding ancestral outliers within each of the 59 populations based on population structure analysis (for details see Methods), we retained 69 242 out of 70 328 pigs genotyped by single-nucleotide polymorphism (SNP) arrays (averaging 45 418 autosomal SNPs) or low-coverage whole-genome sequencing (WGS) (averaging 198 178 autosomal SNPs) for subsequent analyses. This cohort included 20 706 Duroc, 9159 Landrace, 34 540 Yorkshire and 4837 animals from 11 other pig breeds (Table S1). We imputed genotypes for all 69 242 animals to the sequence level using the multi-breed Pig Genomics Reference Panel (PGRP version 1) from the PigGTEx project [34] (Fig. 1b and c). We kept 28 297 602 imputed SNPs with dosage R-squared (DR^2^) > 0.8 and minor allele frequency (MAF) ≥ 0.01 for subsequent analyses (Fig. S1a–e). Through 20 cross-validations, we estimated average concordance rates and Pearson's correlations between imputed and observed genotypes across 14 breeds to be 96.67% and 0.939, respectively (Fig. 1d, Fig. S1f–h, Tables S1–S3). The population structure of GWAS samples, estimated from imputed genotypes, closely matched that from raw genotypes (Pearson's correlation = 0.996), and the distribution of imputed SNPs aligned well with all the SNPs in the genotype reference panel across diverse genomic features (Fig. S1i–l). Altogether, these results supported the reliability of our imputed genotype data.

We collected 271 continuous and 15 binary traits across 59 populations, representing 14 pig breeds worldwide (Fig. S2a). After excluding low-quality phenotypes (Methods), we retained 249 continuous traits and 11 binary traits for subsequent analyses (Table S4). These 260 traits represent 5 **main trait-categories** (with 17 sub trait-categories): **Production** (*n* = 57 612), comprising Feed intake (*n* = 240), Growth (*n* = 57 612) and Feed conversion (*n* = 19 095); **Meat and Carcass** (*n* = 65 883), comprising Fatness (*n* = 60 203), Anatomy (*n* = 52 470), Chemistry (*n* = 368), Fatty acid content (*n* = 368), Texture (*n* = 140), Meat color (*n* = 140) and pH (*n* = 140); **Health** (*n* = 2139), including Immune capacity (*n* = 1317) and Blood parameters (*n* = 2139); **Reproduction** (*n* = 49 018), including Reproductive traits (*n* = 30 207), Litter traits (*n* = 41 907) and Reproductive organs (*n* = 40 049); and **Exterior** (*n* = 6625), including Behavioral (*n* = 2797) and Conformation (*n* = 3828) (Fig. 1e). The average heritability across all traits was 0.27, varying from 0.09 for the number of mummified pigs to 0.91 for lysozyme level (Table S4, Fig. S2b and c). Notably, **Reproduction** traits exhibited a lower heritability (an average of 0.18) compared to **Health** traits (average of 0.50) and **Meat and Carcass** traits (average of 0.32). This aligns with previous studies indicating low heritability for reproductive traits [35–39] and medium to high heritability for health traits [40–45].

### Sharing patterns of QTL between populations revealed by meta-GWAS

We conducted a total of 2117 individual GWAS for the 260 traits across 59 pig populations (Table S4). To ensure the quality and reliability of these individual GWAS results for subsequent meta-analyses, we implemented stringent quality control measures across four different strategies: SE-N (i.e., inverse of the median standard error versus the square root of the sample size) plot, P-Z (i.e., for each SNP, compares the reported P-values with the P-values computed from the Z-statistics based on reported beta-estimate and standard error) plot, effect allele frequencies (EAF) plot and λ_GC_ (Methods) (Fig. S2d–j). This process yielded 2056 high-quality summary-level GWAS results for 232 traits (Table S4), with 78 traits across all 5 main trait-categories not previously included in the Pig QTLdb (release 46) [24] (https://www.animalgenome.org/cgi-bin/QTLdb/SS/index) (Table S5). Generally, Pearson's correlations of SNP effects on the same traits across populations were significantly higher than those on different traits within populations (Fig. S3a and b). We identified 8098 QTL (i.e. 8098 significant trait-associations) (*P* < 5 × 10^−8^), representing 7011 distinct lead SNPs. Among them, 45.66% were detected solely in one population (Methods) (Fig. S3c), where the MAF was higher in the respective populations compared to others (Fig. S3d). For instance, rs323720776, associated with average daily gain (birth-100 kg), was only identified in one Yorkshire population (*n* = 4383), with a MAF of 0.46 versus an average MAF of 0.28 across all 20 studied populations (*n* = 19 824) (Fig. S3e). The findings suggest that differences in the MAF of genetic variants among populations could contribute to population-specific associations.

**Figure 2.**
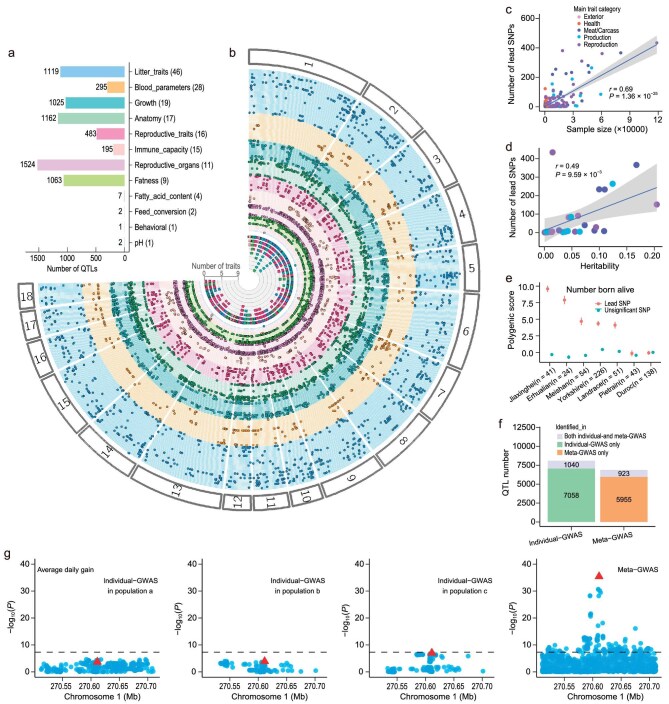
Summary and validation of quantitative trait loci (QTL) for pig complex traits. (a) The total number of QTL detected in 12 sub trait-categories. The number of traits included in each trait-category is shown in parentheses. (b) A Fuji-plot summarizes the 6878 lead SNPs (*P* < 5 × 10^−8^) identified in 169 within-breed and cross-breed meta-GWAS analyses. The plot was created using the Fuji-plot script developed by Kanai *et al.* [85]. The innermost ring (ring 1) indicates the number of traits associated with each lead SNP. Rings 2–170 indicate the 169 meta-GWAS. The order of traits is shown in Table S5 (starting with the innermost ring). The points indicate the genomic position of the 6878 lead SNPs in chromosomes 1–18. The colors represent the trait-category, consistent with (a). (c) Pearson correlation between sample size and the number of lead SNPs in 169 meta-GWAS. (d) Pearson correlation between heritability and the number of lead SNPs in 27 meta-GWAS (sample size > 15 000). Heritability was estimated using linkage disequilibrium score regression (LDSC) [86]. The Pearson correlation coefficient in (c and d) was calculated by the *cor.test* function in R. (e) Results of genomic predictions for individuals from several pig breeds in the PGRP with large phenotype differences, based on a linear mixed model and effect information from suggestive lead variants (*P* < 1 × 10^−5^) and the same number of random non-significant variants in the total number born alive. The x-axis labels indicate the different pig breeds. The y-axis labels indicate the genomic estimated breeding values (GEBVs). The error bars are the standard errors of GEBVs. (f) The number of different categories of QTL detected in individual GWAS and meta-GWAS. Both individual- and meta-GWAS QTL refer to the QTL that exhibit physical position overlap in both individual GWAS and meta-GWAS for the same trait. (g) rs320375241 associated with average daily gain (ADG) in individual GWAS and cross-breed meta-GWAS, respectively. (a), (b) and (c) were three random populations for ADG.

To enhance the detection of QTL and identify shared associations across populations with small effects often overlooked by individual GWAS [46], we conducted cross-breed meta-GWAS for each of the 232 complex traits using the 2056 high-quality GWAS summary statistics (Methods, Table S4). Additionally, we conducted 36 within-breed meta-GWAS analyses for 12 complex traits with large sample sizes within the Duroc, Landrace and Yorkshire breeds (prefixed as ‘D_’, ‘L_’ and ‘Y_’, respectively), to explore potential breed-specific genetic regulatory mechanisms underlying complex traits. In total, we obtained 268 meta-GWAS results, with an average sample size of 10 147 individuals, ranging from 140 for dressing percentage to 58 725 for backfat thickness (BFT) (Table S5). We identified a total of 6878 QTL (*P* < 5 × 10^−8^), representing 6233 distinct lead SNPs (Table S6, Fig. 2a). These lead SNPs were distributed across all 18 autosomes (Fig. 2b) and exhibited a lower MAF compared to random background SNPs in the respective populations (Fig. S4a). The number of QTL detected in both cross-breed and within-breed meta-GWAS showed a positive correlation with sample size (Pearson's *r* = 0.69, *P* = 1.36 × 10^−25^) and trait heritability (Pearson's *r* = 0.49, *P* = 9.59 × 10^−3^) (Fig. 2c and d), aligning with human genetic studies [47,48].

To validate the detected QTL, we implemented three strategies. First, we conducted cross-breed and within-breed meta-GWAS for average daily gain (ADG) across 9 independent populations [49], including 42 790 Duroc pigs, 88 984 Landrace pigs and 69 606 Yorkshire pigs. The association signals identified in these validation populations exhibited significant enrichment (mean fold = 6.84, *P* < 1 × 10^−300^) and replicated an average of 48.49% of the QTL identified in our study (Fig. S4b). Second, we utilized 447 and 379 lead SNPs of suggestive significance (*P* < 1 × 10^−5^) to estimate polygenic scores for the number born alive and teat number, respectively, across 7 pig breeds. Notably, Jiaxinghei, Erhualian and Meishan pigs displayed higher estimated polygenic scores compared to Landrace and Yorkshire (Fig. 2e, Fig. S4c), in line with previous findings that Jiaxinghei, Erhualian and Meishan pigs have greater prolificacy than Landrace and Yorkshire pigs [50–52]. Third, we used lead SNPs (*P* < 5 × 10^−8^) identified from within-breed and cross-breed meta-GWAS of ADG and BFT to perform genomic prediction in 3 independent populations including 93 Duroc, 1510 Landrace and 2844 Yorkshire pigs. We found that the prediction accuracy of the model using lead SNPs from within-breed and cross-breed meta-GWAS was higher than that of the models using lead SNPs (and random SNPs) only from within-breed meta-GWAS (Fig. S4d and e). QTL with higher significance showed a greater overlap with those reported in Pig QTLdb (release 46) [24] (Fig. S4f). These results together underscored the reliability of the detected QTL and indicated a substantial degree of sharing across breeds.

In comparison to individual GWAS results, 5955 out of the 6878 QTL identified in cross-breed and within-breed meta-GWAS were novel across 147 traits (Fig. 2f). For ADG, rs320375241 exhibited non-significance in any individual GWAS but demonstrated significance in cross-breed meta-GWAS (Fig. 2g). In contrast, 7058 out of the 8098 QTL identified in individual GWAS were not replicated in either cross-breed or within-breed meta-GWAS (QTL_individual_GWAS_only_) (Fig. 2f). Lead SNPs of these QTL_individual_GWAS_only_ exhibited a higher proportion of effects in the opposite direction across populations compared to those detected in both individual GWAS and meta-GWAS (Fig. S4g). Our findings suggested that QTL identified in cross-breed and within-breed meta-analyses were more likely to represent shared genetic associations underlying complex traits across populations, in contrast to QTL identified in individual GWAS. Therefore, in subsequent analyses, we focused solely on the 6878 QTL identified in cross-breed and within-breed meta-GWAS to elucidate the genetic regulatory mechanisms underlying complex traits.

### Breed specificity and pleiotropy of QTL

**Figure 3.**
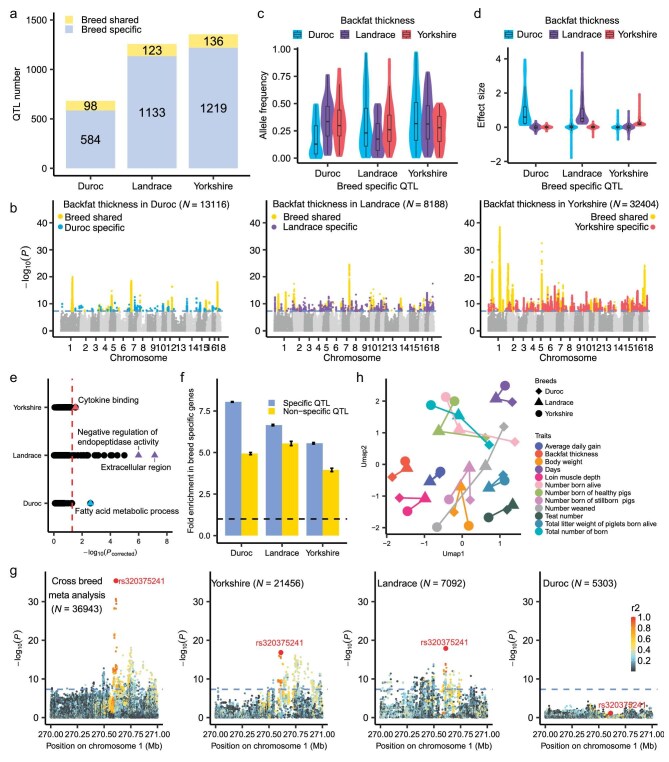
Identified QTL across breeds. (a) The number of QTL from the within-breed meta-analysis for 12 complex traits in Duroc, Landrace and Yorkshire. Breed-specific QTL refers to the QTL that does not exhibit physical overlap with QTL from other breeds for the same trait. Breed-shared QTL refers to the QTL that exhibits physical overlap with QTL from the same trait in at least one breed. (b) Manhattan plots of within-breed meta-analysis for backfat thickness in Duroc (left), Landrace (middle) and Yorkshire (right). The blue (*n* = 68), purple (*n* = 192) and red (*n* = 211) points represent the breed-specific QTL identified in Duroc, Landrace and Yorkshire, respectively. (c and d) The distribution of allele frequencies (c) and effect sizes (d) of lead SNPs of breed-specific QTL for backfat thickness in the three breeds. Boxplots depict the median value as the center, and first and third quartiles as box boundaries. Sample sizes for each breed are provided in Table S5. (e) The Gene Ontology (GO) and Kyoto Encyclopedia of Gene and Genomes (KEGG) enrichment results of breed-specific highly expressed genes in three breeds identified from muscle RNA samples of PigGTEx [34]. The enrichment was conducted using KOBAS [87]. The sample sizes of muscle for detecting breed-specific highly expressed genes in Duroc, Landrace and Yorkshire were 157, 49 and 119, respectively. The x-axis represents the false discovery rate (FDR)-corrected *P*-value. (f) Enrichment of breed-specific QTL and non-breed-specific QTL for loin muscle depth in the breed-specific highly expressed genes in muscle. The error bar represents the standard error of the enrichment fold. (g) Local Manhattan plots of the cross-breed meta-analysis of ADG in all breeds (left 1), as well as a within-breed meta-analysis of Yorkshire (left 2), Landrace (left 3) and Duroc (left 4) on chromosome 1. The color indicates the magnitude of the linkage disequilibrium (LD) between rs320375241 and other SNPs. (h) Clustering for a 36 within-breed meta-analysis of 12 complex traits based on the Z-score of breed shared lead SNPs (lead SNPs with m-values > 0.9 in all the three breeds). Clustering was conducted using the umap package in R.

To explore the breed-specific regulatory effects of genetic variants on complex traits, we first conducted within-breed meta-GWAS analyses for Duroc, Landrace and Yorkshire, respectively, resulting in 3293 QTL in at least one breed across 12 complex traits (Fig. 3a). Of these QTL, 89% exhibited breed-specific effects (see Methods) (Fig. 3a). For instance, we identified 68, 192 and 211 QTL with breed-specific effects for BFT in Duroc, Landrace and Yorkshire, respectively (Fig. 3b). The observation of breed-specific QTL is likely to be a result of the differences in allele frequencies or sample sizes among the three breeds (Fig. 3c and d, Fig. S5) [27]. Additionally, we observed a negative correlation between the effect sizes of lead SNPs and their MAFs across different breeds (Fig. S6a–l). To further investigate the potential regulatory mechanism of breed-specific QTL, we identified genes with specific high expression levels in each of the breeds (breed-specific expressed genes), based on multiple tissue samples (see Methods, Fig. 3e). Enrichment analyses revealed that breed-specific QTL exhibited a higher enrichment in breed-specific expressed genes compared to non-breed-specific QTL present in at least two breeds (Fig. 3f, Fig. S6m–q). These findings suggested that breed-specific effects on complex traits may arise from gene regulatory differences, potentially resulting from gene–environment interactions and genetic drift [53].

The cross-breed meta-analysis replicated 28% of the QTL identified in within-breed meta-analyses across traits (Fig. S6r), while the remaining 72% of QTL often exhibited varying directions of effects for lead SNPs among the three breeds (Fig. S6s–u). Taking ADG as an example, the QTL with lead SNP rs320375241 was consistently identified in the within-breed analyses for Landrace and Yorkshire but not for Duroc, as well as in the cross-breed analysis with increased statistical power (Fig. 3g, Table S6) [27]. Furthermore, utilizing the effect information of breed-shared lead SNPs, the 36 within-breed meta-GWAS summary results can be clustered by traits rather than breeds (Fig. 3h).

We further explored the pleiotropic effects of variants across 232 traits based on the cross-breed meta-GWAS results using PLACO [54]. As expected, we observed a higher proportion of shared effects within the same trait-category compared to those shared between different trait-categories (Fig. S7a, Table S5). Additionally, we estimated the posterior probability of lead SNP effects on each trait in 25 complex traits with the largest sample sizes using METASOFT [55]. Our analysis revealed that 8.24% (174 SNPs) of lead SNPs exhibited an effect on at least two complex traits (m-value > 0.9 from METASOFT). For instance, rs336748894 on chromosome 7 was simultaneously associated with Loin muscle area (*P* = 7.15 × 10^−24^), Loin muscle depth (*P* = 5.87 × 10^−18^), BFT (*P* = 3.26 × 10^−15^), Days (*P* = 2.23 × 10^−6^) and ADG (*P* = 1.59 × 10^−7^) (Fig. S7b). Notably, lead SNP tended to have a smaller effect if it showed effects on more traits (Fig. S7c).

### Functional characterization of GWAS loci by integrating multi-omics data

**Figure 4.**
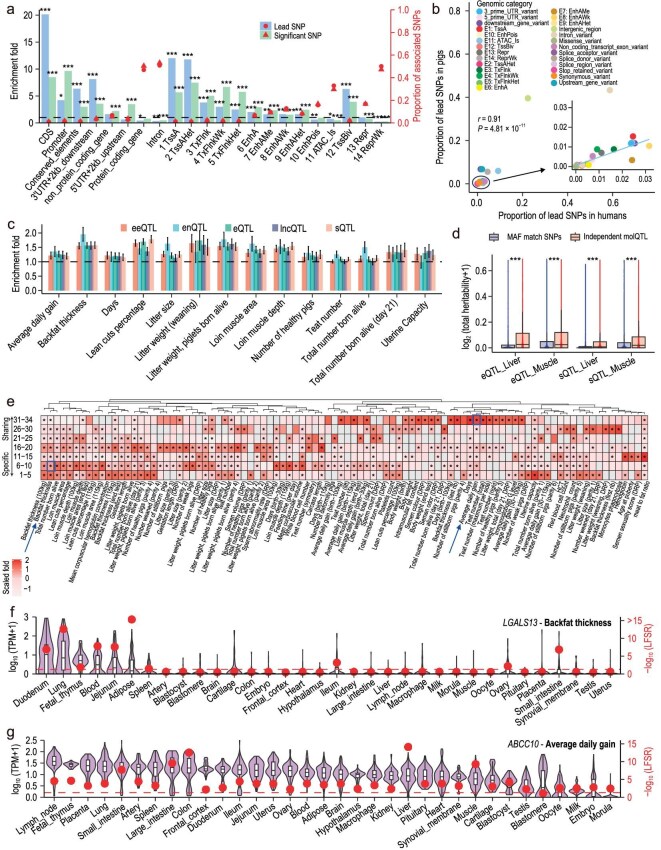
Exploiting the PigGTEx resource to decipher regulatory mechanisms of GWAS loci. (a) Results of annotation and enrichment of lead SNPs and genome-wide-level significant SNPs from within- and cross-breed meta-GWAS with a sample size greater than 1000 in different categories of genomic regions, conserved elements and regulatory elements. The enrichment analyses were conducted using resampled SNPs that matched the MAF (within 0.02) and LD (within 0.1) of the significant SNPs. The red dots and bars indicate the proportion and the enrichment fold of associated SNPs in the category. Significance of enrichment was indicated by *, ** and *** for *P* < 0.05, 0.01 and 0.001, respectively. (b) Annotation results of the lead SNPs identified in this study (y-axis) and downloaded from the human GWAS catalog (https://www.ebi.ac.uk/gwas/) (x-axis) in each category of SNPeff and chromatin states (https://figshare.com/articles/dataset/6_type_of_regulator_hg19_zip/13480425). Each dot represents a genomic category. The Pearson correlation coefficient and significance were calculated by the *cor.test* function in R. (c) The heritability enrichment for 5 types of molecular *cis*-QTL (molQTL) in 14 complex traits with large sample size. The dashed line represents enrichment fold = 1. The error bar reflects the standard error of the enrichment fold. *cis*-eQTL: gene expression QTL, *cis*-sQTL: splicing QTL, *cis*-eeQTL: exon expression, *cis*-lncQTL: lncRNA expression QTL, *cis*-enQTL: enhancer expression QTL. (d) The estimated total SNP heritability contributed by independent molQTL in the liver and muscle for 232 complex traits. Significance is indicated by *** for *P* < 0.001, which was obtained by the *Wolcox.test* of ggsignif package in R. (e) The heritability enrichment for the genes of 7 tissue-sharing gradients in 94 complex traits with a sample size > 1000. The red color represents the scaled heritability enrichment fold. The bright blue boxes indicate the position of examples (f and g). The ‘*’ indicates heritability enrichment fold >1 and *P* < 0.05. Column clusters were produced by the *dist* function with the ‘euclidean’ method and the *hclust* function with the ‘complete’ method in R. The heatmap was plotted by ggplot2 package (v3.3.2) in R (v4.2.1). (f) Expression (left, black) and the overall tissue-sharing pattern at LFSR obtained by MashR (v0.2–6) (right, red) of *LGALS13* in 34 tissues. (g) Expression (left, black) and the overall tissue-sharing pattern at LFSR obtained by MashR (v0.2–6) (right, red) of *ABCC10* in 34 tissues. The red dashed line in (f and g) indicates LFSR < 5%.

To explore the biological context in which the meta-GWAS loci act and their underlying regulatory circuits, we annotated the GWAS loci from both cross-breed and within-breed meta-GWAS using multi-layered biological data, including the functions of genomic variants and 14 different chromatin states previously annotated by Pan *et al.* [56]. We focused on 5837 unique lead SNPs identified in 176 meta-GWAS with sample sizes >1000. Notably, 99.04% of the 5837 lead SNPs were located in non-coding regions (Fig. 4a and Fig. S8a), with 9.41% and 52.73% in promoters and enhancers, respectively. This aligns with previous human studies (Pearson's *r* = 0.91, *P* = 4.81 × 10^−11^) (Fig. 4b). Furthermore, lead SNPs exhibited a significant enrichment in regulatory elements, particularly active promoters (TssA) (12.01-fold, *P* < 1.0 × 10^−30^) and enhancers (EnhA) (2.02-fold, *P* = 1.24 × 10^−5^) (Fig. 4a and Fig. S8b). Notably, the coding sequence (CDS) showed a high enrichment fold (20.16-fold, *P* < 1.0 × 10^−30^), despite only 1.01% of lead SNPs mapping to this region. Additionally, compared to non-significant SNPs, lead SNPs showed a greater density near the transcription start sites (TSSs) of protein-coding genes (Fig. S8c) and lower evolutionary constraints across species (lower PhastCons scores, Fig. S8d).

To further investigate the regulatory role of genetic variants on complex traits, we integrated molQTL corresponding to five molecular phenotypes (molPhe, including cis-eQTL for protein-coding genes expression, cis-eeQTL for exon expression, cis-lncQTL for lncRNA expression, cis-enQTL for enhancer expression and cis-sQTL for alternative splicing) from 34 tissues in PigGTEx [34]. We performed summary-based heritability enrichment analyses for these molQTL in cross-breed and within-breed meta-GWAS with sample size exceeding 1000, and detected 296 significantly enriched molQTL-trait pairs across 73 meta-GWAS (false discovery rate, FDR < 0.05) (Fig. S8e, Table S7). In general, at least one type of molQTL exhibited significant enrichment for trait heritability in 14 out of the 25 complex traits with large sample sizes (FDR < 0.05, mean enrichment fold = 1.37 ± 0.21) (Fig. 4c, Table S7). We further explored the heritability contributions of independent eQTL and sQTL to complex traits and found that they explained a higher proportion of heritability compared to MAF-matched SNPs in muscle and liver tissues (Fig. 4d). These results suggested that regulatory variants contributed to dissecting genetic and molecular architecture underlying complex traits in pigs.

Furthermore, we observed that different trait categories exhibited heritability enrichment across eGenes (genes with at least one significant variant in at least one tissue) with distinct tissue-sharing patterns (Table S8, Fig. 4e). For instance, BFT (fatness of sub trait-category) showed a notable enrichment for tissue-specific eGenes, while ADG (growth of sub trait-category) was enriched in tissue-sharing eGenes (Fig. 4e). The lead SNP (rs1108824455) of BFT was identified as an eQTL that regulates the expression of *LGALS13*, a gene with specific expression in a few tissues, including the duodenum, lung and adipose (Fig. 4f, Fig. S8f). *LGALS13* has been recognized as a serum biomarker during early pregnancy [57,58]. In contrast, the lead SNP (rs324200444) of ADG was also an eQTL that regulated the expression of *ABCC10*, a gene displaying extensive tissue sharing regarding both gene expression and eQTL (Fig. 4g, Fig. S8g). *ABCC10* is known to act as a genetic marker for pig growth [59].

### Detecting trait-relevant tissues

**Figure 5.**
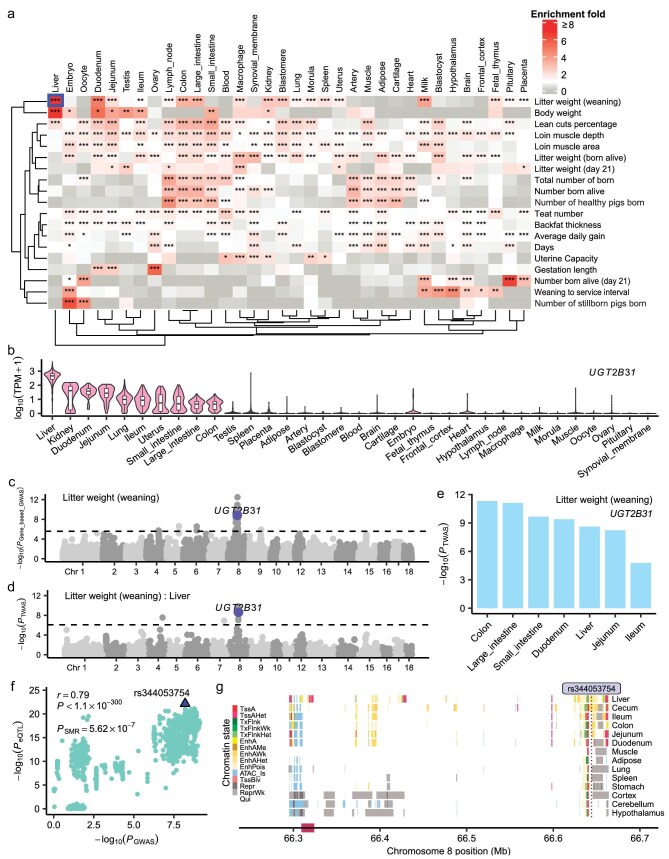
Tissue-specific regulation of GWAS loci. (a) Enrichment results for significantly associated SNPs of 19 cross-breed meta-GWAS in tissue-specific functional regions (the top 1000 tissue-specific highly expressed genes per tissue and their upstream and downstream 100 kb regions) in 34 tissues. Colors indicate enrichment fold. Rows indicate traits and columns indicate tissues. The bright blue box indicates the position of examples (b–g). Enrichment for trait-tissue pairs *E*_T_ = *p*_T_ (proportion of significant SNPs for trait *Tr* located in functional regions of tissue *Ti*)/*q*_T_ (proportion of all SNPs located in functional regions of tissue *Ti*), as calculated with BEDTools v2.25.0 [88]. Associated SNPs were resampled 1000 times with MAF within 0.02 and LD within 0.1, matched to calculate enrichment significance. *E*_T_ > 1 and *P* < 0.05 indicates the significant trait-tissue pair. Significance of enrichment is indicated by *, ** and *** for *P* < 0.05, 0.01 and 0.001, respectively. (b) Expression of gene *UGT2B31* in 34 tissues. (c) A Manhattan plot representing the gene-based GWAS results of litter weight (weaning). (d) A Manhattan plot representing the single-tissue TWAS results of litter weight (weaning) in the liver. (e) The single-tissue TWAS results of *UGT2B31* for litter weight (weaning) from S-PrediXcan [89]. (f) The summary data-based Mendelian randomization (SMR) results of litter weight (weaning) GWAS (x-axis) and *cis*-eQTL (y-axis) of *UGT2B31* on chromosome 8 in the liver. (g) The chromatin states around *UGT2B31* on chromosome 8.

To further investigate the tissue-mediated patterns of genetic regulation of complex traits, we conducted enrichment analyses for meta-GWAS signals within tissue-specific expressed genes across 34 pig tissues (Table S9, Fig. S9). Trait-associated SNPs were significantly enriched in tissue-specific genes of trait-relevant tissues (Fig. 5a and Table S10). For example, both litter weight (weaning) (M_TLWT_Weaning) (10.14-fold, *P* = 0.001) and body weight (M_BW) (6.84-fold, *P* = 0.001) exhibited the highest enrichment in the liver compared to other tissues. Likewise, gestation length exhibited the highest enrichment in the ovary (5.62-fold, *P* = 0.001) (Fig. 5a).

Based on the results of the transcriptome-wide association study (TWAS), summary-based mendelian randomization analysis (SMR) and co-localization analyses [34] conducted on within-breed and cross-breed meta-GWAS with a sample size greater than 1000, we identified 56 003 variant-molPhe-tissue-trait circuits, including 14 829 variant-gene-tissue-trait regulatory circuits (Table S11). For instance, *UGT2B31*, which exhibits the highest expression in the liver compared to other tissues (Fig. 5b), was significantly associated with litter weight (weaning) through gene-based GWAS (*P* = 1.48 × 10^−9^) (Fig. 5c) and TWAS (*P* = 2.46 × 10^−9^) (Fig. 5d and e). Furthermore, a *cis*-eQTL (rs344053754) regulated litter weight (weaning) through influencing *UGT2B31* mRNA expression in the liver (*P* = 5.62 × 10^−7^, SMR) (Fig. 5f). The SNP rs344053754 is located in active enhancers in the liver and intestines, but not in other tissues (Fig. 5g). *UGT2B31* is an important drug metabolizing enzyme in the liver across various animal species [60–63]. In summary, these analyses provided many testable biological hypotheses of gene regulation underlying complex traits for functional follow-ups.

### Exploring shared genetic regulation of complex traits between pigs and humans

**Figure 6.**
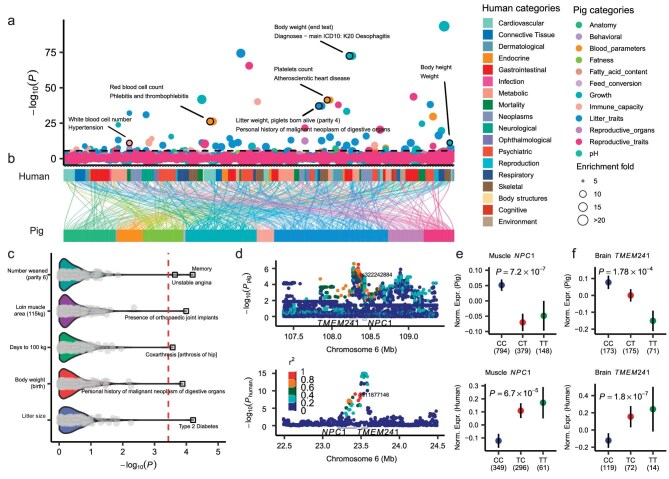
Comparison of complex trait genetics between humans and pigs. (a) The heritability enrichment of 126 pig GWAS in 136 human GWAS. The x-axis indicates traits and trait categories of human GWAS. The y-axis indicates the significance of heritability enrichment. The color of the dots indicates the trait category of pig GWAS. The size of the dots indicates the fold of heritability enrichment. The dashed line indicates the significant threshold (*P* = 0.05/126*136 pig traits). (b) An alluvium-stratum plot showing the correlation between human and pig GWAS. Colors indicate trait categories. (c) Spearman's correlations of traits between humans and pigs, which were estimated by the absolute Z score of homologous variants from GWAS summary statistics. The x-axis indicates the significance of Spearman's correlations. The red dashed line indicates the significant threshold (*P* = 0.05/136 human traits). The top trait pairs are labeled. (d–f) Similar regulatory mechanisms between body fatness rate (BFR) in humans and ADG in pigs. (d) A local Manhattan plot of GWAS for ADG in pigs (top) and BFR in humans (bottom). The red triangles represent homozygous variants of humans (rs11877146) and pigs (rs322242884). The color indicates the magnitude of LD between the homologous variants and other SNPs. (e) The effects of homozygous variants in (d) on the expression of homozygous gene *NPC1* in the muscle of pigs (top) and humans (bottom). (f) The effects of homologous variants in (d) on the expression of the homologous gene *TMEM241* in the brain of pigs (top) and humans (bottom). The black error bars in (e and f) represent the standard errors. The significance tests in (e and f) were performed with the *wilcox.test* function of the *ggsignif* package in R (v4.2.1). The effect size of eQTL in humans and pigs in (e and f) was derived from the GTEx portal (https://www.gtexportal.org/) and PigGTEx portal (https://piggtex.farmgtex.org/), respectively.

To explore the shared genetic regulatory mechanisms underlying complex traits between humans and pigs, we conducted heritability enrichment analyses on pig GWAS loci from each of the 126 pig meta-GWAS with a sample size exceeding 1000, in relation to orthologous QTL (*P* < 5 × 10^−8^) from 136 human diseases/traits across 18 trait domains (Table S12). These analyses revealed 62 significant human–pig trait pairs (enrichment fold > 1 and *P* < 0.05/126*136), representing 21 pig traits and 47 human traits (Fig. 6a and b, Table S13), including body weight (end test) in pigs and esophagitis in humans (enrichment fold = 8.30, *P* = 3.13 × 10^−73^) (Fig. 6a and b). For these 62 human–pig trait pairs, the corresponding conserved, homologous and random loci exhibited lower but conserved pig GWAS loci showing a similar heritability and enrichment fold (Fig. S10, Table S13). We also calculated Spearman's correlations for human–pig trait pairs based on the absolute *Z*-score (effect size) of homologous variants from GWAS summary statistics. We observed significant correlations for physiologically relevant traits between the two species (Fig. 6c, Table S14). For example, the Days to 100 kg in pigs showed a significant correlation with Coxarthrosis in humans (Spearman's *r* = −0.07, *P* = 2.70 × 10^−4^), litter size in pigs correlated with type 2 diabetes in humans (Spearman's *r* = 0.10, *P* = 6.16 × 10^−5^), and body weight at birth in pigs correlated with personal history of malignant neoplasm of digestive organs in humans (Spearman's *r* = 0.08, *P* = 1.32 × 10^−4^) (Fig. 6c). These findings complement established discoveries in human research [64–67]. Meanwhile, we introduced novel insights, such as the number weaned in pigs is correlated with memory and unstable angina in humans, and loin muscle area in pigs is associated with the presence of orthopedic joint implants in humans (Fig. 6c). Furthermore, we identified potential conserved regulatory mechanisms between humans and pigs. For example, rs322242884, associated with ADG in Landrace pigs (*Z*-score = −4.53, *P* = 5.80 × 10^−6^), has a homologous variant rs11877146 associated with body fat percentage in humans (*Z*-score = 6.06, *P* = 1.33 × 10^−9^) (Fig. 6d). Notably, these homologous variants were eQTLs for *NPC1* in the muscle (*P_human_* = 6.70 × 10^−5^, *P_pig_* = 7.20 × 10^−7^), and for *TMEM241* in the brain (*P_human_* = 1.80 × 10^−7^ and *P_pig_* = 1.78 × 10^−4^) in both species (Fig. 6e and f). Consistently, previous studies have proposed that *NPC1* was associated with body weight and adipocyte processes across various animals [68–71] and *TMEM241* was associated with bone degeneration and osteoporosis [72]. These results together provided evidence for shared regulatory mechanisms underlying complex traits between humans and pigs, supporting the use of pigs as models for studying human biology and diseases.

## DISCUSSION

In summary, to maximize the power of QTL discovery in pigs, we harmonized population-scale data sets from 59 diverse populations, encompassing a total of 70 328 pigs. We then carried out a large-scale cross-breed meta-GWAS analysis for 232 complex traits and within-breed meta-GWAS for 12 complex traits, representing 22 production traits, 54 meat and carcass traits, 61 health traits, 89 reproduction traits and 6 exterior traits (Fig. 1e). In total, we reported 6878 QTL for 169 traits. We annotated most of these QTL by examining regulatory elements from the FAANG project [33] and molQTL from the PigGTEx project [34]. We obtained 14 829 variant-gene-tissue-trait circuits, in which regulatory variants affect complex traits by modulating gene expression in specific tissues. Finally, by comparing with 136 publicly available human GWAS summary statistics, we revealed a certain conservation of genetic and regulatory mechanisms of complex traits between pigs and humans. Our findings provided evidence for using pigs as biomedical models to investigate the underlying genetic mechanisms of human biology and diseases [19,73], particularly for those with challenging phenotypic accessibility in humans.

While the QTLdb has documented 48 875 QTL from 426 publications on pig complex traits [24], the applicability of these findings across populations/breeds is limited as most of them were derived from individual populations. The multi-ancestry meta-analyses in humans have shown increased statistical power for detecting novel and shared genetic associations [23,26–28]. To the best of our knowledge, in the present study, we generated the largest-scale genetic diversity resource to date for studying complex traits in pigs. Our results demonstrated that cross-breed meta-GWAS analysis enhances the transferability of predictions across breeds [28]. In addition, by comparing within-breed meta-analyses in Duroc, Landrace and Yorkshire, we identified 2936 breed-specific QTL for 12 complex traits, including 3 production and 7 reproduction traits. The breed-specific lead SNPs tended to exhibit lower allele frequencies and higher effect sizes in the discovered breed than in other breeds. This aligns with human research and may be partly a result of the difference in sample size [27]. Our findings also implied that breed-shared QTL provide greater insight into the genetic architecture of traits, as breed-shared lead SNPs can cluster GWAS results according to traits rather than breeds. Conversely, we observed a higher proportion of QTL exhibiting pleiotropy within the same trait category compared to between trait categories, implying that multi-trait joint analysis could elucidate complex regulatory mechanisms shared among traits, as indicated in [74].

Functional annotation of GWAS results facilitates the identification of putative causal variants and guides subsequent empirical bench-based approaches to elucidate the molecular and cellular mechanisms behind GWAS loci [75,76]. By examining multi-omics data and functional annotation of various genomic features, we found that GWAS loci were significantly enriched in regulatory elements, including promoter and enhancer, as well as regulatory variants, such as eQTL and sQTL (QTL for alternative splicing). We observed distinct gene regulation patterns of complex traits. Specifically, the backfat thickness of fatness exhibited a significant enrichment in tissue-specific genes. On the other hand, the average daily gain of growth showed enrichment in ubiquitously expressed genes. This is in line with a previous study that demonstrated changes in mitochondrial DNA content and mitochondrial gene expression levels across multiple tissues during growth [77]. Furthermore, we proposed that rs344053754 has an impact on litter weight at weaning through influencing the expression of *UGT2B31* in the liver. Nevertheless, further validation of this hypothesis necessitates functional laboratory research. These findings provide valuable insights for the design of subsequent functional experiments and the advancement of animal breeding biotechnology. We also recognized several limitations within the current study. First, the ample sizes for many traits are modest, leading to reduced statistical power for QTL discovery. In the future, we will increase the sample size for these traits by combining data from more populations via meta-GWAS analyses. In addition, we will explore the impact of other types of genomic variants, such as copy number variants and InDels (insertions and deletions), on molecular and complex traits by employing long-read sequencing and pangenome data. Although our integrative analysis has shed light on the genes and variants potentially involved in the underlying mechanisms of complex traits, future functional validation remains essential.

## MATERIALS AND METHODS

### Data collection and processing

We collected genotype and phenotype data in 70 328 pigs from 59 study populations, representing 14 pig breeds (details in Table S1). The phenotypes included 286 complex traits categorized into Reproduction, Meat and Carcass, Health, Production and Exterior (details in Table S4). To ensure data consistency, we implemented a standardized pipeline for processing individual-level genotype and phenotype data across populations. Ultimately, we retained 28 297 603 unique SNPs in 69 242 individuals, encompassing 20 706 Duroc, 34 540 Yorkshire, 9159 Landrace and 4837 from other pig breeds. Additionally, we retained 260 complex traits for downstream analyses (see Supplementary Data for details).

### Individual GWAS and quality control

We conducted individual GWAS for each trait in each population. We performed association analysis using a logistic mixed model with fastGWA-GLMM in GCTA (v1.94.0) [78] for binary traits, and using a mixed linear model with fastGWA in GCTA (v1.94.0) [79] for quantitative traits. The model for each trait might be different for each population, but in general, we considered farms, sex, year and season as fixed effects and included the first five genotype principal components from within each population after removing outliers as covariates when necessary. Specifically, for 49 traits that have phenotypic records in multiple time points for the same individual (e.g. sperm traits and litter sizes, detailed in Table S4), we employed MMAP (v2021_08_19_22_30.intel) (https://mmap.github.io/) to perform association analysis based on their de-regressed proofs and weights calculated by DMU software (v6-R5-2-EM64T) [80]. Details are provided in Supplementary Data.

To enable individual GWAS from different populations to be comparable in the meta-analysis, we checked all summary-level GWAS results based on EasyQC [81]. After stringent quality control procedures, we ultimately retained 2056 out of 2117 individual GWAS for subsequent analysis, involving 232 complex traits. Details are provided in Supplementary Data.

### Meta-analysis of GWAS

To identify the QTL shared across populations, as in [23], we performed cross-breed meta-analyses on the filtered individual GWAS results for each of the 232 traits using METAL (v2011-03-25) [82]. In addition, we performed 36 within-breed meta-analyses for 12 complex traits with large sample sizes in Duroc, Landrace and Yorkshire, respectively. In addition, we conducted gene-based GWAS for each trait using MAGMA (v1.10) [83]. Details are provided in Supplementary Data.

### Definition and validation of QTL

For both individual GWAS and meta-GWAS, we used *P* < 5.0 × 10^−8^ as the genome-wide significance threshold and defined lead SNPs and QTL based on genomic position by referring to the research of cattle stature [23]. If a QTL for trait A overlaps but does not fully coincide with a QTL for trait B, they are counted as two separate QTL, i.e. two trait-associations. To validate the identified QTL regions, we used three strategies. First, we validated the QTL regions in independent populations. Second, we used information on suggestive lead SNPs (*P* < 1.0 × 10^−5^) for breed-level genomic prediction. Third, we tested genomic prediction in the independent populations. Details are provided in Supplementary Data.

## Supplementary Material

nwaf048_Supplemental_Files

## Data Availability

The summary statistics of cross-breed and within-breed meta-GWAS underlying this article are available in ScienceDB (https://www.scidb.cn/en/s/ZRjEve), and can be queried and visualized in the PigBiobank web portal (https://pigbiobank.farmgtex.org/). The integrated results of meta-GWAS and molQTL from PigGTEx, such as TWAS, SMR and co-localization, are also available in ScienceDB (https://www.scidb.cn/en/s/zU7rIj). The BioProject of WGS samples in the genotype imputation reference panel PGRP are listed in Supplementary Table 2. Chromatin states of pigs are available through the UCSC Genome Browser (http://genome.ucsc.edu/s/zhypan/susScr11_15_state_14_tissues_new). The raw genotype and phenotype data of 14 populations downloaded from the public database are shown in Supplementary Table 15. The raw genotype and phenotype data of the remaining populations are not publicly available since they are commercial breeding data, but they are available from the corresponding author upon reasonable request. The codes for genotype imputation, GWAS, functional enrichment, heritability enrichment and cross-species comparison are stored on GitHub (https://github.com/SCAU-AnimalGenetics/Pig-metaGWAS). Scripts and code for co-localization, SMR and TWAS are available at the FarmGTEx GitHub website (https://github.com/FarmGTEx/PigGTEx-Pipeline-v0).
