# Supplementary material for: Integrating large-scale meta-GWAS and PigGTEx resources to decipher the genetic basis of 232 complex traits in pigs
Source: Natl Sci Rev. 2025 Feb 17;12(5):nwaf048. doi: 10.1093/nsr/nwaf048 (PMC12051865; doi:10.1093/nsr/nwaf048)
Supplement: nwaf048_Supplemental_Files [file nwaf048_supplemental_files.zip › Supplementary file final.docx]

**Methods**

**Ethics**

This is not applicable because no biological samples were collected, and no animal handling was performed for this study.

**Data collection and processing**

*GWAS dataset*. In total, we collected 70,328 pigs with genotype and phenotype data from 59 study populations (including 14 public populations) covering 14 pig breeds (Table S1). We conducted comprehensive data preparation and standardization for phenotype and genotype, respectively. The details are as follows.

*Genotype data preprocessing.* These 70,328 pigs were genotyped using genotyping arrays, including the Illumina Porcine SNP60K Bead Chip (N = 10,870), the GeneSeek Genomic Profiler (GGP) Porcine SNP80 BeadChip (N = 4,724), the GGP Porcine SNP50 BeadChip (N = 29,789), the KPS Porcine Breeding Chip (N = 21,618), and the GenoBaits Porcine SNP50K BeadChip (N = 454), or low-coverage sequence (N = 2,873). We constructed a standard pipeline to uniformly process individual-level genotype data for all 59 populations. Briefly, we first converted the coordinate of the genomic version of genotype data to the Sscrofa11.1 (v100) and only kept the autosomal biallelic SNPs. To identify and remove outliers within each population, we performed principal component analysis (PCA) within each of the 59 populations using PLINK (v1.9) [1] based on LD-independent SNPs with parameters: “*--mind 0.1 --geno 0.1 --maf 0.01 --indep-pairwise 50 5 0.5 --pca 10*”. We visualized the principal components (PCs) for each population in R (v3.4.3) and excluded a total of 1,086 outliers using PLINK (v1.9). Finally, we retained 69,242 individuals for downstream analyses, including 20,706 Duroc pigs, 34,540 Yorkshire pigs, 9,159 Landrace pigs and 4,837 other pigs.

*Genotype imputation.* To obtain genotype data at the whole-genome sequence (WGS) level, we performed genotype imputation for each population based on multi-breed Pig Genomics Reference Panel (PGRP v1) from PigGTEx [2], which consists of 42,523,218 autosomal biallelic SNPs from 1,602 WGS samples covering over 100 pig breeds (Table S2). We firstly removed duplicate alleles from array data using PLINK (v1.9) [1] with parameter: “*--list-duplicate-vars ids-only suppress-first, --exclude plink.dupvar --recode vcf bgz*” and kept biallelic SNPs using BCFtools (v1.9) [3]. We then employed conform-gt program (<http://faculty.washington.edu/browning/conform-gt.html>) to revise strand inconsistencies of SNPs [4]. To check the genetic structure both the PGRP v1 and the GWAS populations (after removing within-population outliers), we merged all the GWAS individuals with PGRP v1 individuals and performed PCA based on 1,603 shared SNPs using PLINK (v1.9). We checked the genetic structure by visualizing the first two PCs in R (v3.4.3). We imputed the genotype data of target populations to sequence level using Beagle (v5.1) [5] and filtered out variants with dosage R-squared (DR^2^, the estimated squared correlation between the estimated and the true allele dose) < 0.8 and MAF < 0.01 within each population. Finally, we retained a total of 28,297,603 unique SNPs across all 59 populations for downstream analysis (Table S1).

*Imputation accuracy evaluation.* To evaluate the accuracy of genotype imputation, we employed two strategies (Fig. S1a). (1) We conducted 20 times of five-fold cross-validation using genotype data of 60,720 samples from 53 GWAS populations that had individual-level genotype data. Specifically, in each round of cross-validation, we randomly masked 20% of SNPs in chromosome 6 of the target panel and imputed them with the remaining unmasked SNPs using PGRP as a reference panel via Beagle (v5.1). (2) We conducted an external validation using 65 WGS samples from NCBI that were independent of PGRP, comprising of 35 Duroc pigs (PRJNA712489) and 30 Suhuai pigs (PRJNA791712) (Table S3). First, we employed Trimmomatic (v0.39) [6] to filter out the adaptors and low-quality reads, mapped clean reads to Sus scrofa11.1 (v100) using BWA-MEM (v0.7.5a-r405) with default parameters [7], and marked duplicated reads using Picard (v2.21.2) (<http://broadinstitute.github.io/picard/>). We then called SNPs for these samples using Genome Analysis Toolkit (GATK) (v4.1.4.1) [8]with parameter: “*QD> 2, MQ < 40, FS > 60, SOR > 3, MQRankSum < -12.5* and *ReadPosRankSum < -8*”, resulting in 17,182,138 and 15,696,890 biallelic autosomal SNPs for Duroc and Suhuai, respectively. To evaluate the accuracy of genotype imputation, we kept the same SNPs in the array and then imputed them to WGS level using PGRP as reference panel via Beagle (v5.1). Finally, we measured the imputation accuracy by calculating the concordance rate and Pearson’s correlation between imputed genotypes with DR2 > 0.8 and MAF > 0.01 and true genotypes in the validation sets.

*Phenotype data collection.* A total of 286 complex traits (15 binary traits and 271 continuous traits) were available for the 59 populations (Table S4), which belonged to five main trait-categories (i.e., Reproduction, Meat and Carcass, Health, Production, and Exterior) and 17 sub trait-categories (i.e., Litter, Reproductive, Growth, Reproductive organs, Blood parameters, Immune capacity, Anatomy, Fatness, Fatty acid content, Feed conversion, Conformation, Meat color, Chemistry, Feed intake, pH, Texture, and Behavioral).

*Phenotype data preprocessing.* In particular, 49 out of 286 traits have phenotypic records in multiple time points for the same individual (e.g., sperm traits and litter sizes, detailed in Table S4) and were referred to as “multiple time points trait” (i.e., MT-trait). For these 49 MT_traits, we calculated the de-regressed proofs (DRP) using DMU (v6-R5-2-EM64T) [9]. Specifically, we first estimated breeding values (EBV) in each population based on pedigree information using a single-trait repeatability model implemented in the DMUAI module of DMU. To eliminate the bias from relatives, we then calculated the DRP and weights for each pig using the methods described by Garrick et al. [10]. We used the DRP and weights for each pig for the downstream association analysis. Before conducting the GWAS, we performed quality control on the phenotypic data. Regarding reproductive traits, we only retained phenotypes from the first six litters. Finally, we retained a total of 260 traits (249 continuous traits and 11 binary traits) for further analysis.

**GWAS**

*Individual GWAS and quality control*. We conducted individual GWAS for each trait in each population as described below and referred to this as “individual GWAS” throughout the manuscript (Table S4). We performed association analysis using a logistic mixed model with fastGWA-GLMM in GCTA (v1.94.0) [11] for binary traits, and using a mixed linear model with fastGWA in GCTA (v1.94.0) [12] for quantitative traits. The model for each trait may be different for each population, but in general, we considered farms, sex, year, and season as fixed effects and included the first five principal components as covariates when necessary. The principal components were derived from PCA conducted within the testing population. Specifically, for 49 MT-traits, we employed MMAP (v2021_08_19_22_30.intel) (<https://mmap.github.io/>) to perform association analysis based on their DRP and weights for each pig.

To enable individual GWAS from different populations to be comparable in the meta-analysis, we checked all summary-level GWAS results based on EasyQC [13]. First, to detect issues related to trait transformations, we examined the relationship between the inverse of the median standard error of all SNPs beta estimates and the square root of the sample size (SE-N plot) across multiple study files for each trait. For outliers, we corrected the raw phenotype data and reran the association analysis. The calibration factor c of the SE-N plot was approximated from the autosomal SNPs of the PGRP reference panel as $c\sim median\left( \frac{1}{\sqrt{2\mathrm{MAF}_{j}\left( 1-{MAF}_{j} \right)}} \right)$. Second, we examined the analytical issues for each study by comparing the reported *P* values of each SNP with the *P* values computed from the *Z*-statistics (*Z*-statistics = $\beta_{j}/SE{(\beta)}_{j}$) based on reported beta estimate and standard error (P-Z plots). Third, we plotted the effect allele frequency (EAF) from the study population against EAF from PGRP to identify strand issues or allele miscoding that could severely reduce statistical power. Fourth, we grasped the potential problems with population stratification by computing the genomic control (GC) inflation factor (λ_GC_, from 0.86 to 2.39 with an average of 1.11). After we reconstructed the association analyses using the first five principal components as additional covariates, the λ_GC_ decreased (from 0.56 to 1.58 with an average of 1.04). Fifth, we excluded SNPs with missing or nonsensical information (e.g., *P* values < 0 or >1, or non-numeric values such as “NA”) from summary results. Finally, we retained 2,056 high-quality individual GWAS for subsequent analysis.

*Meta-analysis of GWAS*. To identify the QTL shared across populations, as in [14], we performed meta-analyses on the filtered individual GWAS results for each trait using METAL (v2011-03-25) [15], based on an inverse variance-weighted fixed effects model that weights effect size estimates according to estimated standard errors and allows for different population frequencies of genotypes and alleles. Genomic control correction was applied for all input files in the analysis. SNPs included in the meta-analysis were present in at least one individual GWAS, and the total number of SNPs for each trait is shown in Table S5. In addition, we conducted gene-based GWAS for each trait using MAGMA (v1.10) [16]. In which, we annotated SNPs to Ensembl (Sscrofa11.1 v100) genes and used the PGRP as the reference for LD. We considered 0.05/N as the significance threshold, where N is the effective number of genes involved in each gene-based analysis.

**Definition and** **validation of QTL**

*QTL definition*. For both individual GWAS and meta-GWAS, we used *P* < 5.0 × 10^-8^ as the genome-wide significance threshold and defined lead SNPs and QTL on the basis of genomic position referred to the study on cattle stature [14]. For each GWAS summary, we defined the significant SNP with the smallest *P*-value in each chromosome as the first lead SNP, and defined the position where the first non-significant SNP appeared upstream and downstream of the first lead SNP as the boundary of the first potential QTL region. We defined the significant SNP with the smallest *P*-value outside the first potential QTL region as the second lead SNP, and defined the position where the first non-significant SNP appeared upstream and downstream of the second lead SNP as the boundary of the second potential QTL region. This process was iterated until there were no significant SNPs on the chromosome. Finally, we reported the two most distant significant SNPs within 0.5 Mb on both sides of the lead SNP in each potential QTL region as the boundaries of the final QTL. If a final QTL for trait A overlaps but does not fully coincide with a final QTL for trait B, they are counted as two separate QTL. If trait A and trait B share the same lead SNP, it is counted as one lead SNP.

*QTL validation*. To validate the identified QTL regions, we used the following three strategies.

First, we validated the QTL regions in independent populations. For this, we downloaded the average daily gain (ADG) of three Duroc (N=42,790), three Landrace (N=88,984), and three Yorkshire (N=69,606) pig populations [17]. We performed individual GWAS on these nine populations using the MLMA model of GCTA (v1.94.0) [18], and conducted within-breed meta-GWAS analyses and across-breed meta-analysis using METAL [15]. We identified QTL regions using the same method as in this study. We calculated the enrichment fold for these QTL regions in QTL regions detected from our ADG meta-analysis.

Second, we used information on suggestively lead SNPs (*P* < 1.0 × 10^-5^) for breed-level genomic prediction to validate the functional reliability of the QTL. For this, we performed genomic predictions in seven pig breeds from PGRP, including 54 Meishan, 24 Erhualian, 41 Jiaxinghei, 226 Yorkshire, 51 Landrace, 138 Duroc, and 43 Pietrain pigs. We extracted the genotypes of the lead SNPs from PGRP using Bcftools (v1.9) [3]and extracted their effect sizes from GWAS summary statistics. Whereafter, we used a linear mixed model to fit the genotype and effect size for genomic prediction in each breed. The model formula we used for each breed was:

$$y=\sum_{i=1}^{M} Z_{i}g_{i}$$

where $y$ is a vector of predicted phenotypes, $g_{i}$ is the effect size of lead SNP $i$ in GWAS summary statistics, $Z_{i}$ is the vector of the genotype of lead SNP $i$ containing 0, 1 and 2. We fitted the model using R v 4.2.1. For comparison, we randomly selected the same number of SNPs and performed the same analysis.

Third, we test genomic prediction in the independent populations including 93 Duroc, 1,510 Landrace, and 2,844 Yorkshire pigs. We applied the same genotype imputation pipeline and quality control standards to impute genotypes for these three populations from 51,797 SNPs to the PGRP v1, resulting in 9,665,885, 9,791,894, and 11,249,921 SNPs for Duroc, Landrace and Yorkshire pigs, respectively. We corrected phenotypic values ($y_{c}$) for each individual within each population using a linear model $y_{c}=y-X\hat{b}$. Where $y_{c}$ is the vector of corrected phenotypes, $y$ is the vector of raw phenotypes, $\hat{b}$ is the vector of fixed effects, including testing herd, year and season of testing and sex. $X$ is the design matrix for fixed effects. $X\hat{b}$ was calculated through ‘lm’ function in R. We then extract lead SNPs identified from within- and cross-breed meta-GWAS for average daily gain (ADG) and backfat thickness (BFT), and randomly selected SNPs with *P* > 0.5 from the meta-GWAS summaries, for genomic prediction with the following model:

$$y_{c}=1\mu+Zg+e$$

where $y_{c}$ is the vector of corrected phenotypes, $1$ is the vector of ones, $\mu$ is the overall mean, $g$ is the vector of genetic effects with its design matrix $Z$, $e$ is the vector of residuals. The $g$ and $e$ are assumed to be normally distribution: $g\sim N(0, \sigma_{g}^{2}G)$ and $e\sim N(0, \sigma_{e}^{2}I)$ , where $\sigma_{g}^{2}$ and $\sigma_{e}^{2}$ are the additive genetic variance and residual variance, respectively, and $I$ is the identity matrix. The $G$ matrix was constructed using LDAK (v5.2) software with a parameter that calculate kinship matrix in one step: *--calc-kins-direct*. We used a 10 × 5-fold cross-validation to evaluate the prediction accuracy. The genomic prediction accuracy was determined by calculating Pearson correlations between the predicted estimated breeding value and the corrected phenotypes.

Furthermore, we compared the QTL regions based on physical location for those from the same traits reported in the Pig Quantitative Trait Locus (QTL) database (Pig QTLdb version 46) [19] (<https://www.animalgenome.org/cgi-bin/QTLdb/SS/index>). We excluded downloaded QTL with missing start/end position information and QTL regions smaller than 1bp or larger than 1Mp. This resulted in a final retention of 302,784 autosomal QTL regions. which matched 151 out of our 232 traits.

**Detection of breed-specific and breed-shared QTL and** **pleiotropic variants across traits**

To identify breed-specific and breed-shared detected QTL, we performed a comparison for QTL regions detected from within-breed meta-GWAS of 12 complex traits conducted in Duroc, Landrace and Yorkshire pigs. We defined QTL that did not overlap in physical location between breeds for the same trait as breed-specific QTL, and QTL that overlap across breeds by at least 1 bp as breed-shared QTL. We fine-mapped and reported a 95% credible set for each breed-specific and breed-shared QTL using SuSiE [20]. To explore the potential regulatory mechanism of breed-specific QTL, we detected the top 100 breed-specific highly expressed genes based on the normalized expression (Transcripts Per Million, TPM) in muscle samples (157 Duroc, 49 Landrace, and 119 Yorkshire) and liver samples (5 Duroc, 56 Landrace, and 62 Yorkshire) from PigGTEx. We analyzed the function of these breed-specific genes by Gene Ontology (GO) term analysis and Kyoto Encyclopedia of Gene and Genomes (KEGG) pathway analysis using KOBAS (<http://kobas.cbi.pku.edu.cn/kobas3>) [21], with a significance of Bonferroni-adjusted *P*-Value < 0.05. We then performed enrichment analysis for breed-specific QTL and non-breed-specific QTL in breed-specific highly expressed genes, with a permutation test by resampling genes 1,000 times. The enrichment fold was calculated by the number of overlaps between breed-specific QTL and/or non-breed-specific QTL and breed-specific genes/the number of overlaps between breed-specific QTL and/or non-breed-specific QTL and randomly selected genes (the same number as breed-specific genes). Furthermore, we downloaded samples of liver, gluteus medius, and duodenum from previous study [22], each tissue containing 100 Duroc, 100 Landrace, and 100 Yorkshire, respectively. We performed the same enrichment analysis for these three tissues with their related traits, which identified from the section ‘Enrichment of GWAS loci in tissues’ below.

In addition, we extracted the effect sizes and standard errors of lead SNPs, and used METASOFT (v2.0.1) [23], a procedure that corrects for the effect of sample size on association analysis, to calculate the posterior probability (m-value) of the lead SNP effect on each trait in each breed and effects on multiple traits. We considered an m-value greater than 0.9 as evidence of an effect. We also conducted a pleiotropy GWAS for each pair of 232 traits on a variant-level based on all lead SNPs from individual-GWAS and meta-GWAS using PLACO software [24]. To investigate whether the effects of lead SNPs detected in individual GWAS were shared across populations, we similarly calculated the m-value of each lead SNP effect on the traits in each population using the METASOFT software. To ensure the reliability of our results, we only considered non-MT traits with study populations ≥ 5.

**Annotation and enrichment of significant/lead variants in functional categories**

To investigate the molecular mechanisms of significant/lead SNPs, we examined multiple layers of biological data. Here we only consider significant/lead SNPs identified from 176 traits with sample size > 1,000.

First, we annotated significant and lead SNPs in several genomic categories: (i) 20 genomic variant categories, including intron variants and intergenic region variants, annotated using SnpEff (v.4.3) [25]. (ii) the seven genomic regions categorized by genomic locations concerning protein-coding genes, i.e., CDS, promoter (100 kb upstream and downstream of the protein-coding gene TSS), 5’UTR + 2 kb upstream, 3’UTR + 2 kb downstream, protein-coding genes, non-protein-coding genes, and intron regions. (iii) the downloaded mammalian conserved elements identified from Multiple Sequence Alignments (MSA) using the Genomic Evolutionary Rate Profiling (GERP) software based on 103 mammals (<https://ftp.ensembl.org/pub/release-100/bed/ensembl-compara/103_mammals.gerp_constrained_element/>). (iv) the 14 chromatin states representing regulatory function detected from 14 major pig tissues [26].

Second, we estimated the enrichment for significant and lead SNPs across the various genomic categories. For genomic variants, we used the *oddsratio* function of fmsb (v0.7.5) package [27] in R (v4.1.2) to perform enrichment and estimate significance. The enrichment for category *C* (*E*_C_) = *p*_C_ (proportion of significant and lead SNPs located in category *C*) / *q*_C_ (proportion of all SNPs located in category *C*). For the genomic regions of protein-coding genes, conserved elements, and chromatin states, we perform enrichment using BEDTools (v2.25.0)[28]. The enrichment for category *C* (*E*_C_) = *p*_C_ (proportion of category *C* in all significantly enriched trait-category pairs *E*_T_) / *q*_C_ (proportion of category *C* in the genome). Here, the enrichment for trait-category pairs *E*_T_ = *p*_T_ (proportion of significant and lead SNPs for trait *T* located in category *C*)/*q*_T_ (proportion of all SNPs located in category *C*). We performed a permutation test by resampling the association signals 10,000 times to determine if the observed number of SNPs located in the annotation category was greater than what would be expected by chance, using the R package regioneR (v1.24.0) [29]. Additionally, we resampled the SNPs matching the MAF (within 0.02) and LD (*r*^2^ within 0.1) of the association signals 1,000 times for the permutation test. An *E*_C_ greater than one and a *P*-value less than 0.05 indicated that significant and lead SNPs were significantly enriched in category *C*.

In addition, to understand the evolutionary sequence conservation of association variants, we downloaded PhastCons scores for 100 vertebrate species from UCSC (<http://hgdownload.cse.ucsc.edu/goldenpath/hg38/phastCons100way/hg38.100way.phastCons/>). We converted the Wiggle files of PhastCons scores to BED files using the BEDOPS tool (v2.4.40) [30], and then we lifted them over from the human genome 38 (h38) to Sscrofa11.1 using UCSC's LiftOver tool [31].

**Genetic parameter estimation and heritability enrichment**

*Genetic parameter estimation*. To estimate the genetic parameters for pig complex traits, we used individual-level data and summary-level data, respectively. For individual-level data, we estimate the genetic parameters using GCTA (v1.94.0) with “--reml” parameter. For summary-level data, we first converted all 268 GWAS summary data to the .sumstats file using the *munge_sumstats.py* script from the linkage disequilibrium score regression (LDSC v1.0.1) [32], with the parameters: “--sumstats --N --out”, and estimated linkage disequilibrium (LD) score for SNPs from PGRP using the *ldsc.py* script with the parameters: “--l2, --ld-wind-kb 1000” for each chromosome. Second, we estimated the narrow-sense heritability for complex traits based on summary using LDSC (v1.0.1) with parameters: “--h2, --ref-ld-chr, --w-ld-chr”.

*Heritability enrichment.* To investigate the impact of regulatory variants on complex traits, we extracted significant *cis*-molQTL from five molecular phenotypes in 34 tissues, including 2,930,627 *cis*-eQTL for protein-coding gene expression, 2,842,703 *cis*-eeQTL for exon expression, 2,628,257 *cis*-sQTL for alternative splicing, 2,703,774 *cis*-enQTL for enhancer and 2,056,718 *cis*-lncQTL for lncRNA [2]. We performed heritability enrichment analysis for these five type of molQTL on 176 meta-GWAS with sample size > 1,000 based on the BLD-Thin model of LDAK (v5.0) [33] with an optional parameter: “-*-check-sums NO*”. Furthermore, we compared the genetic contribution of independent molQTL and random SNPs to complex traits by estimating their respective SNP heritability using LDAK.

**Enrichment of GWAS loci in tissues**

To investigate the genetic contribution of genes with *cis*-eQTL in different tissue-sharing level for complex traits, we categorized protein-coding genes into seven groups (1-5, 6-10, 11-15, 16-20, 21-25, 26-30, 31-34 tissues) from tissue-specific to tissue-sharing, based on the *cis*-eQTL of 34 tissues from PigGTEx [2]. Briefly, we performed a meta-analysis of the top *cis*-eQTL across all 34 tissues using MashR (v0.2-6) [34]. We defined a *cis*-eQTL with the significance levels (that is, local false sign rate (LFSR) < 0.05) as active in a given tissue. We found an average of 9,503 eGenes (genes with at least one eQTL in the tissue) in 34 tissues. To control the effect of sample size differences across tissues, we extracted the top 9,503 genes with the smallest LFSR in each tissue. We grouped the number of shared tissues for a gene based on how many tissues had an LFSR < 0.05 for the *cis*-eQTL of the gene. We then conducted a heritability enrichment analysis for each eGene group in 176 GWAS summaries which sample size > 1,000 using LDAK (the BLD-Thin model) [33]. In this, we randomly selected 500,000 variants for annotation file of each gene group.

To explore the potential tissue involved in the genetic mechanisms of complex traits, we detected the tissue-specific expressed genes for 34 tissues from FarmGTEx [2] and performed enrichment analysis. Firstly, we referred to the method proposed by Finucane et al. [35], which accounts for sample size and other factors on differential expression detection between tissues. We calculated a *t*-statistic for specific expression for each gene in each tissue versus all other tissue samples, respectively, based on the gene expression of 34 tissues. We ranked genes in each tissue by the *t*-statistic and defined the top 1,000 genes with the highest *t*-statistic to be the tissue-specific expressed genes. We then added a 100-kb window upstream and downstream of the genes to get a genome annotation. We analyzed the function of these tissue-specific genes by GO term analysis and KEGG pathway analysis using KOBAS (<http://kobas.cbi.pku.edu.cn/kobas3>) [21]. The pathways or annotations with Bonferroni-adjusted *P*-Value < 0.05 were regarded as significant results. Secondly, we used BEDTools (v2.25.0) to test whether the significant SNPs of each complex trait were significantly enriched in the genome annotation of each tissue. The enrichment for trait-tissue pairs *E*_T_ = *p*_T_ (proportion of significant SNPs for trait *T* located in genome annotation for tissue *t*)/*q*_T_ (proportion of all SNPs located in genome annotation for tissue *t*). We performed a permutation test by resampling SNPs matching the MAF (within 0.02) and LD (*r*^2^ within 0.1) of the association signals 1,000 times to determine if the observed number of SNPs located in the genome annotation was greater than what would be expected by chance, using the R package regioneR (v1.24.0). An *E*_T_ greater than 1 and a *P*-value less than 0.05 indicated that significant SNPs for trait *T* were significantly enriched in the genome annotation for tissue *t,* and we considered tissue *t* to be the functionally relevant tissues of trait *T*.

**Integrative analysis of GWAS loci and molQTL**

To detect the potential genetic association between molecular phenotypes and complex traits, we employed three analyses including colocalization, TWAS, and Mendelian randomization (MR) as detailed in PigGTEx [2].We performed a colocalization analysis of molQTL and GWAS loci using fastENLOC (v1.0) [36], and defined the regional colocalization probability > 0.9 as significant colocalizations. We conducted single- and multi-tissue TWAS using S-PrediXcan [37] and S-MultiXcan in MetaXcan (v0.6.11) [38], respectively, based on summary statistics from meta-GWAS. We applied the Bonferroni correction for multiple tests and considered a corrected *P*-value < 0.05 to be significant. We conducted MR analysis using the SMR tool (v1.03) with molQTL as instrumental variables [39]. We applied the Benjamini-Hochberg adjusted *P*_SMR_ < 0.05 and *P*_GWAS_ < 1×10^-5^ as significant.

**Comparison of genetic regulation for complex traits between humans and pigs**

*Heritability enrichment analyses*. To investigate whether the regulatory mechanisms of complex traits were conserved between humans and pigs, we performed heritability enrichment analyses for pig GWAS loci from each pig GWAS on each human complex trait. Initially, we downloaded diverse public summary-level GWAS results for 136 human complex traits/diseases involving 18 trait domains with large sample sizes from various repositories, including the International Genomics of Alzheimer's Project and UK Biobank. The sample sizes across these 136 GWAS summaries ranged from 13,239 to 1,232,091, with an average of 387,922. These datasets encompassed individuals of European, African, and Latino ancestry, etc. We then converted the genome coordinates of ±1 Mb regions around lead SNPs of pig GWAS loci to the human genome (GRCh38/hg38) using UCSC’s LiftOver tool [31], where we only considered 126 pig meta-GWAS results which with significant loci and sample size > 1,000. Subsequently, we implemented a heritability enrichment analysis for orthologous pig GWAS loci on the orthologous QTL (*P* < 5 × 10^-8^) of 136 human GWAS using LDSC (v1.0.1) [32]. We considered human-pig trait pairs with heritability enrichment fold > 1 and *P*-value < 0.05/126*136 = 2.92×10^-6^ as significant. To determine the reliability of the heritability enrichment of orthologous pig GWAS loci in human complex traits, we extracted orthologous pig GWAS loci from the conserved regions annotated in 120 mammals with human as reference (downloaded from <https://bds.mpi-cbg.de/hillerlab/120MammalAlignment/Human120way/>) (conserved pig GWAS loci), and randomly selected an equal number and length of regions as those of orthologous pig GWAS loci from the orthologous non-pig GWAS loci within the human genome (orthologous loci), from the conserved regions annotated in 120 mammals with human as reference (downloaded from <https://bds.mpi-cbg.de/hillerlab/120MammalAlignment/Human120way/>) (conserved loci), and from the entire human genome (random loci). We conducted heritability enrichment analyses on these orthologous loci, conserved loci and random loci using the same methods applied to the pig GWAS loci.

C*orrelation on GWAS summaries*. To explore the correlation between pigs and humans using GWAS summary statistics, we first obtained homozygous variants shared between pigs (version: Sus scrofa11.1) and human (GRCh38/hg38) using LiftOver [31]. Second, we matched these homozygous variants for 126 pig meta-GWAS with significant loci and sample size > 1,000 and 136 human GWAS. Third, we performed a Spearman correlation test on the absolute Z-score values of the homozygous variants obtained from humans and pigs using R v4.2.1. We employed a statistical significance threshold of 0.05/136=3.68×10^-4^ for the trait pairs in each pig trait, adjusted by Bonferroni correction as did in Shirai Y, *et al*. [40].

**References**

1. Chang CC, Chow CC, Tellier LC *et al.* Second-generation PLINK: rising to the challenge of larger and richer datasets. *Gigascience* 2015;**4**:7.

2. Teng J, Gao Y, Yin H *et al.* A compendium of genetic regulatory effects across pig tissues. *Nat Genet* 2024;**56**:112–23.

3. Li H, Handsaker B, Wysoker A *et al.* The Sequence Alignment/Map format and SAMtools. *Bioinformatics* 2009;**25**:2078–9.

4. Browning SR, Browning BL. Rapid and Accurate Haplotype Phasing and Missing-Data Inference for Whole-Genome Association Studies By Use of Localized Haplotype Clustering. *Am J Hum Genet* 2007;**81**:1084–97.

5. Browning BL, Zhou Y, Browning SR. A One-Penny Imputed Genome from Next-Generation Reference Panels. *Am J Hum Genet* 2018;**103**:338–48.

6. Bolger AM, Lohse M, Usadel B. Trimmomatic: a flexible trimmer for Illumina sequence data. *Bioinformatics* 2014;**30**:2114–20.

7. Li H, Durbin R. Fast and accurate short read alignment with Burrows–Wheeler transform. *Bioinformatics* 2009;**25**:1754–60.

8. McKenna A, Hanna M, Banks E *et al.* The Genome Analysis Toolkit: A MapReduce framework for analyzing next-generation DNA sequencing data. *Genome Res* 2010;**20**:1297–303.

9. Madsen P, Sørensen P, Su G et al. DMU - a package for analyzing multivariate mixed models. 2006:2014:27-11.

10. Garrick DJ, Taylor JF, Fernando RL. Deregressing estimated breeding values and weighting information for genomic regression analyses. *Genet Sel Evol* 2009;**41**:55.

11. Jiang L, Zheng Z, Fang H *et al.* A generalized linear mixed model association tool for biobank-scale data. *Nat Genet* 2021;**53**:1616–21.

12. Jiang L, Zheng Z, Qi T *et al.* A resource-efficient tool for mixed model association analysis of large-scale data. *Nat Genet* 2019;**51**:1749–55.

13. Winkler TW, Day FR, Croteau-Chonka DC *et al.* Quality control and conduct of genome-wide association meta-analyses. *Nat Protoc* 2014;**9**:1192–212.

14. Bouwman AC, Daetwyler HD, Chamberlain AJ *et al.* Meta-analysis of genome-wide association studies for cattle stature identifies common genes that regulate body size in mammals. *Nat Genet* 2018;**50**:362–7.

15. Willer CJ, Li Y, Abecasis GR. METAL: Fast and efficient meta-analysis of genomewide association scans. *Bioinformatics* 2010;**26**:2190–1.

16. de Leeuw CA, Mooij JM, Heskes T *et al.* MAGMA: Generalized Gene-Set Analysis of GWAS Data. Tang H (ed.). *PLOS Comput Biol* 2015;**11**:e1004219.

17. Cai Z, Christensen OF, Lund MS *et al.* Large-scale association study on daily weight gain in pigs reveals overlap of genetic factors for growth in humans. *BMC Genomics* 2022;**23**:133.

18. Yang J, Lee SH, Goddard ME *et al.* GCTA: A tool for genome-wide complex trait analysis. *Am J Hum Genet* 2011;**88**:76–82.

19. Hu Z-L, Park CA, Reecy JM. Bringing the Animal QTLdb and CorrDB into the future: meeting new challenges and providing updated services. *Nucleic Acids Res* 2022;**50**:D956–61.

20. Wang G, Sarkar A, Carbonetto P *et al.* A Simple New Approach to Variable Selection in Regression, with Application to Genetic Fine Mapping. *J R Stat Soc Ser B Stat Methodol* 2020;**82**:1273–300.

21. Bu D, Luo H, Huo P *et al.* KOBAS-i: intelligent prioritization and exploratory visualization of biological functions for gene enrichment analysis. *Nucleic Acids Res* 2021;**49**:W317–25.

22. Crespo-Piazuelo D, Acloque H, González-Rodríguez O *et al.* Identification of transcriptional regulatory variants in pig duodenum, liver, and muscle tissues. *Gigascience* 2022;**12**, DOI: 10.1093/gigascience/giad042.

23. Han B, Eskin E. Interpreting Meta-Analyses of Genome-Wide Association Studies. Kerr K (ed.). *PLoS Genet* 2012;**8**:e1002555.

24. Ray D, Venkataraghavan S, Zhang W *et al.* Pleiotropy method reveals genetic overlap between orofacial clefts at multiple novel loci from GWAS of multi-ethnic trios. Cordell HJ (ed.). *PLOS Genet* 2021;**17**:e1009584.

25. Cingolani P, Platts A, Wang LL *et al.* A program for annotating and predicting the effects of single nucleotide polymorphisms, SnpEff. *Fly (Austin)* 2012;**6**:80–92.

26. Pan Z, Yao Y, Yin H *et al.* Pig genome functional annotation enhances the biological interpretation of complex traits and human disease. *Nat Commun* 2021;**12**, DOI: 10.1038/s41467-021-26153-7.

27. M N. fmsb: Functions for Medical Statistics Book with some Demographic Data. 2019:2020-03–12.

28. Quinlan AR, Hall IM. BEDTools: a flexible suite of utilities for comparing genomic features. *Bioinformatics* 2010;**26**:841–2.

29. Gel B, Díez-Villanueva A, Serra E *et al.* regioneR: an R/Bioconductor package for the association analysis of genomic regions based on permutation tests. *Bioinformatics* 2016;**32**:289–91.

30. Neph S, Kuehn MS, Reynolds AP *et al.* BEDOPS: high-performance genomic feature operations. *Bioinformatics* 2012;**28**:1919–20.

31. Navarro Gonzalez J, Zweig AS, Speir ML *et al.* The UCSC Genome Browser database: 2021 update. *Nucleic Acids Res* 2021;**49**:D1046–57.

32. Bulik-Sullivan BK, Loh P-R, Finucane HK *et al.* LD Score regression distinguishes confounding from polygenicity in genome-wide association studies. *Nat Genet* 2015;**47**:291–5.

33. Speed D, Holmes J, Balding DJ. Evaluating and improving heritability models using summary statistics. *Nat Genet* 2020;**52**:458–62.

34. Urbut SM, Wang G, Carbonetto P *et al.* Flexible statistical methods for estimating and testing effects in genomic studies with multiple conditions. *Nat Genet* 2019;**51**:187–95.

35. Finucane HK, Reshef YA, Anttila V *et al.* Heritability enrichment of specifically expressed genes identifies disease-relevant tissues and cell types. *Nat Genet* 2018;**50**:621–9.

36. Pividori M, Rajagopal PS, Barbeira A *et al.* PhenomeXcan: Mapping the genome to the phenome through the transcriptome. *Sci Adv* 2020;**6**, DOI: 10.1126/sciadv.aba2083.

37. Barbeira AN, Dickinson SP, Bonazzola R *et al.* Exploring the phenotypic consequences of tissue specific gene expression variation inferred from GWAS summary statistics. *Nat Commun* 2018;**9**:1–20.

38. Barbeira AN, Pividori M, Zheng J *et al.* Integrating predicted transcriptome from multiple tissues improves association detection. Plagnol V (ed.). *PLOS Genet* 2019;**15**:e1007889.

39. Zhu Z, Zhang F, Hu H *et al.* Integration of summary data from GWAS and eQTL studies predicts complex trait gene targets. *Nat Genet* 2016;**48**:481–7.

40. Shirai Y, Nakanishi Y, Suzuki A *et al.* Multi-trait and cross-population genome-wide association studies across autoimmune and allergic diseases identify shared and distinct genetic component. *Ann Rheum Dis* 2022;**81**:1301–12.

**Supplementary Figures and legends**


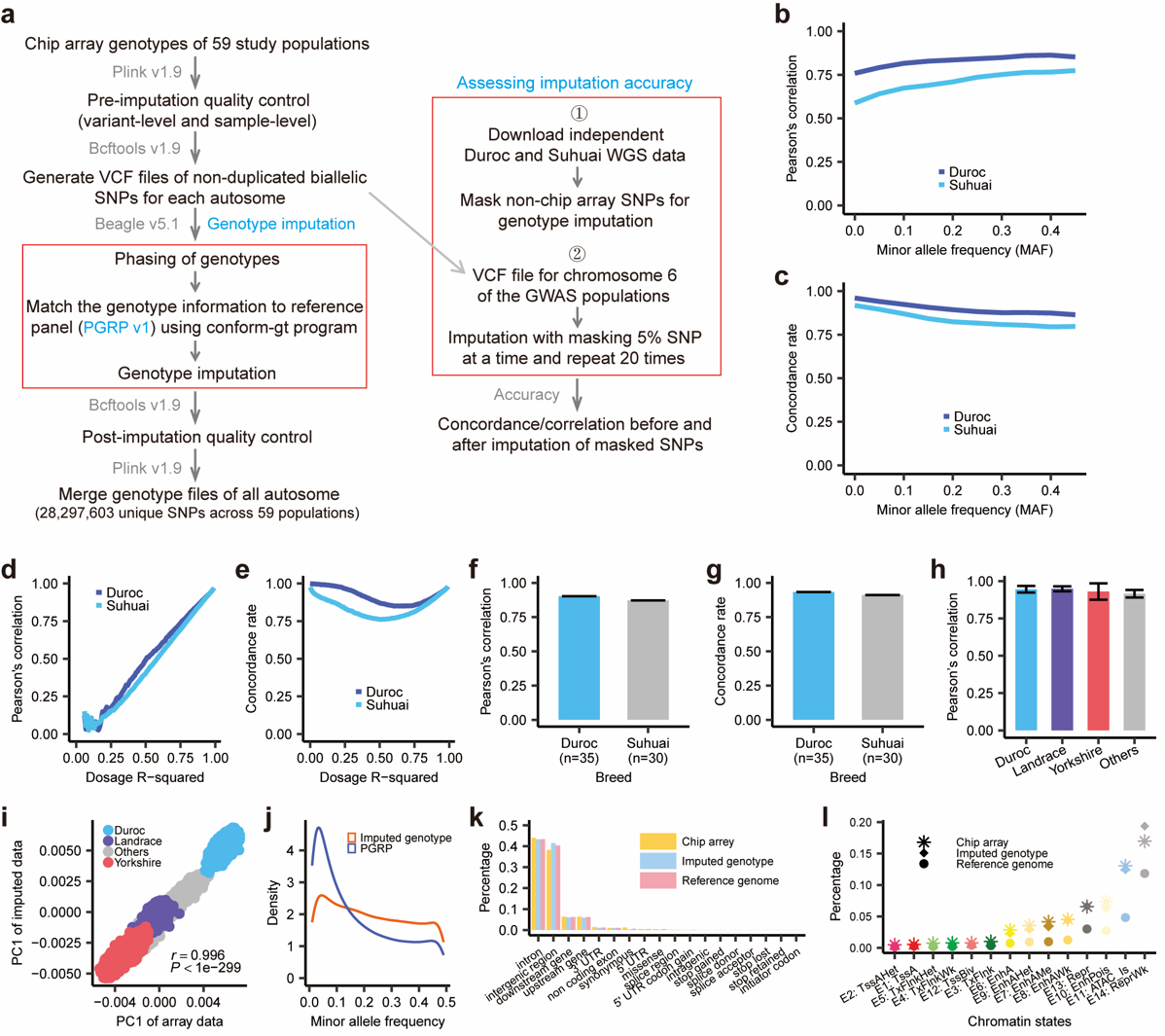


**Figure S1.** **Accuracy assessment of genotype imputation.**

(**a**) Workflow for genotype imputation and imputation accuracy assessment. **(b-e)** The Pearson’s correlation **(b, d)** and concordance rate **(c, e)** between the imputed and true genotypes for independent whole-genome sequencing (WGS) data. **(f-g)** The imputation accuracy of the multi-breed Pig Genomics Reference Panel (PGRP version 1) in independent WGS data, which was calculated as the Pearson’s correlation **(f)** and concordance rate **(g)** between the imputed and true genotypes. Error bar indicates the standard error. The Pearson’s correlation was 90.21% ± 11.51% for Duroc pigs (commercial breed and within PGRP) and 87.22% ± 16.15% for Suhuai pigs (domesticated breed and outside PGRP). The concordance rate was 93.34% ± 7.64% for Duroc pigs and 91.13% ± 10.49% for Suhuai pigs. **(h)** The Pearson’s correlation between the imputed and true genotypes, which were evaluated on chromosome 6 with 20 times of five-fold cross-validation using Chip array data from 60,720 pigs in 53 genome-wide association analysis (GWAS) populations that had individual-level genotype data. The error bars indicate standard deviation. **(i)** The comparison of principal component analysis of GWAS samples using genotypes before and after genotype imputation. The Pearson correlation coefficient and significance was calculated by the *cor.test* function in R. **(j)** The distribution of minor allele frequencies (MAF) for genotypes obtained from PGRP and imputed genotypes from GWAS populations. **(k-l)** Percentage of 28,297,603 unique high quality imputed SNPs vs the whole genome (Pearson’s correlation = 1.0, *P* = 1.1 × 10^-29^) and chip array SNPs (Pearson’s correlation = 0.998, *P* = 1.68 × 10^-21^) across 19 genomic regions annotated by SnpEff (v4.3) [25] **(k)** and 14 chromatin states across 14 pig tissues [26], with Pearson’s correlation = 0.973 (*P* = 5.75 × 10^-9^) for the whole genome and Pearson’s correlation = 0.992 (*P* = 3.46 × 10^-12^) for chip array SNPs **(l)**.


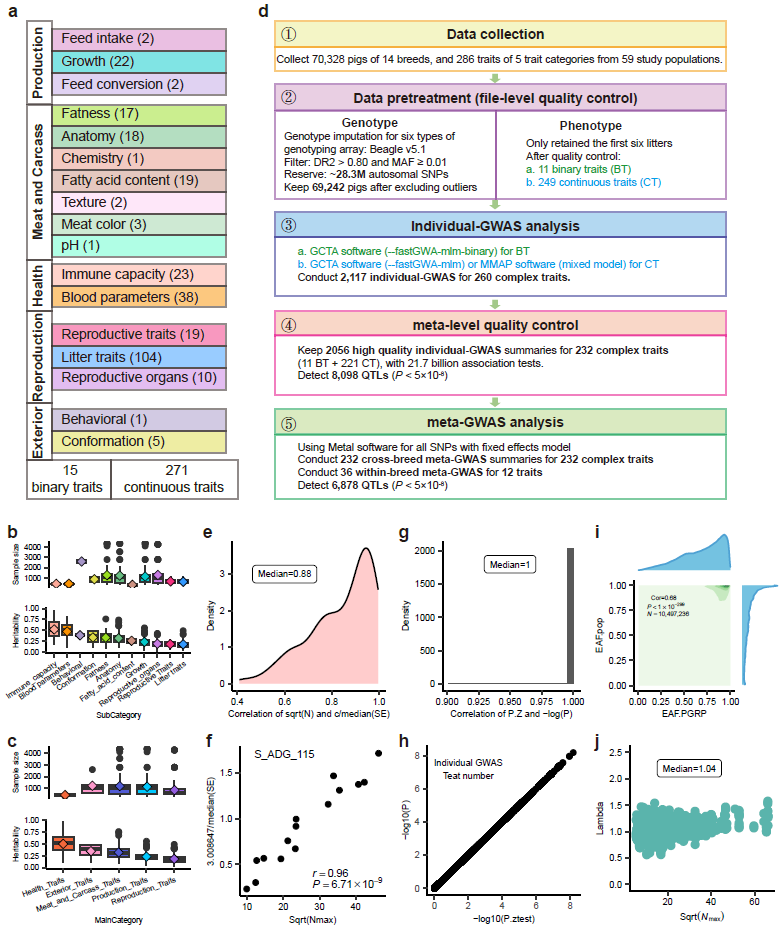


**Figure S2. High quality of individual GWAS.**

**(a)** Summary of collected traits before quality control. A total of 15 binary traits and 271 continuous traits, representing five main trait-categories (left, no colored background) and 17 sub trait-categories (right, with a colored background). **(b-c)** Boxplot of sample size and heritability for 494 individual GWAS with sample size > 300 and *P*-value of heritability < 0.05, which categorized by sub trait-categories in **(b)** and main trait-categories in **(c)**. The diamond represent means. **(d)** Workflow from data collection to meta-GWAS analysis. **(e)** Density of the Pearson’s correlations between the inverse of median standard error of the beta estimates and the square root of the maximum sample size across all SNPs in the individual GWAS summaries from each of 117 traits (with number of individual GWASs > 6 and *P*-values for Pearson correlations < 0.05). N: sample size. c=3.008647. SE: standard error. **(f)** The SE-N plot of average daily gain (birth-115kg) (S_ADG_115) for example, involving 15 individual-GWASs. **(g-h)** P-Z plot that compare *P*-values reported in the association result with *P*-values calculated from Z-statistics (*P*.ztest) derived from the reported effect size and standard error. Density of Pearson’s correlation of the two types of *P* for all individual-GWASs **(g)** and an example in one individual-GWAS file of teat number (left) (TNUM_L) **(h)**. **(i)** The effect allele frequency (EAF) in an individual GWAS population against the genotype imputation reference panel. The Pearson’s correlation coefficient and significance in **(f, i)** was calculated by the *cor.test* function in R. **(j)** Lambda-N plot of 1,759 individual-GWASs without considering MT-traits.


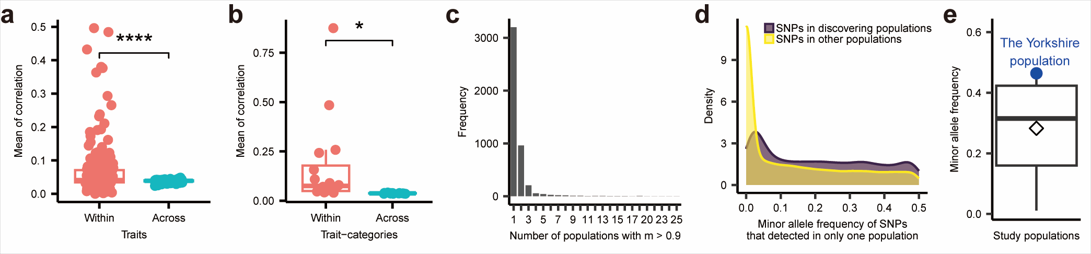


**Figure S3. Lead SNPs identified from individual GWAS.**

**(a)** Average of absolute values of Pearson’s correlations between individual-GWAS pairs for the same traits from different populations and between pairs for different traits. **(b)** Average of absolute values of Pearson’s correlations between individual-GWAS pairs within the same trait-categories and between pairs across trait-categories. Correlations between GWAS pairs in **(a-b)** were calculated using Z-score, based on 79,895 overlap SNPs in all 2,056 individual-GWAS. Significance of differences between groups were estimated using the *t.test* function in R (v4.1.2). **(c)** Distribution of population numbers in which the same trait-SNP association was detected (m-value > 0.9, METASOFT) in individual GWASs. We only consider non-MT-traits with >=5 populations. **(d)** MAF distribution of SNPs that detected association in only one population, where the purple is the MAF distribution of SNPs in the discovering populations, the yellow is the MAF distribution of SNPs in other populations. **(e)** MAF distribution of rs323720776 in all 20 detected populations for Average daily gain (birth-100kg), which association were detected only in one Yorkshire population (blue point). The diamond indicates the mean MAF of *rs323720776* in all 20 study populations.


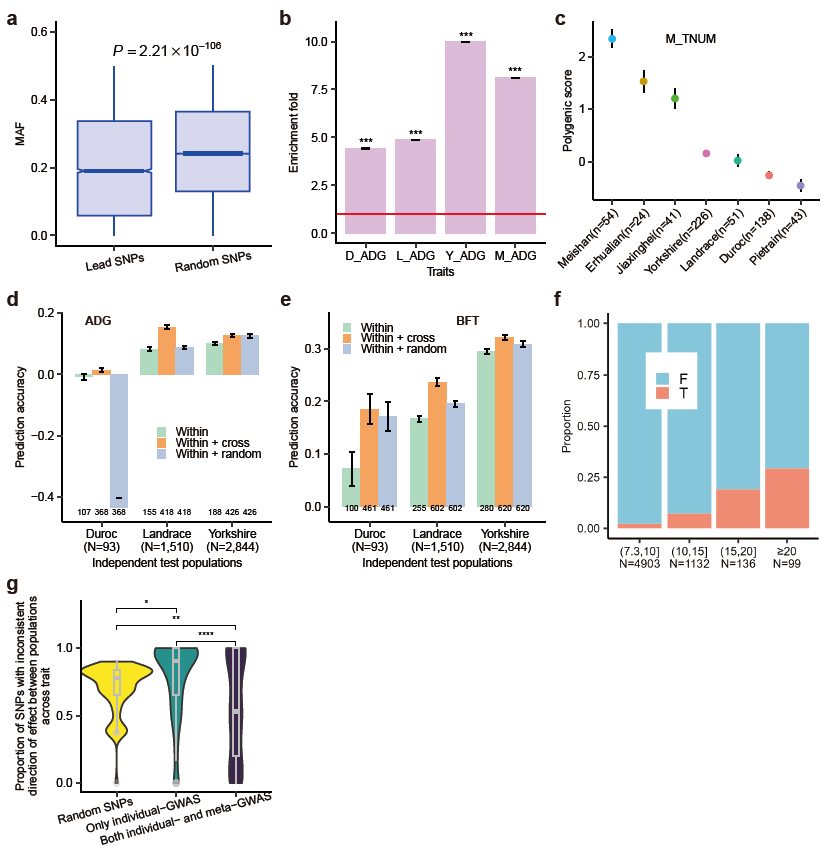


**Figure S4. Lead SNPs and QTL identified from within-breed and cross-breed meta-GWAS.**

**(a)** MAF distribution of lead SNPs and randomly selected SNPs from within-breed and cross-breed meta-GWAS. The random SNPs were selected from each meta-summary with the same number of all lead SNPs. **(b)** Enrichment results of QTL regions (*P* < 1×10^-5^) from independent within-breed and cross-breed meta-GWAS in the QTL regions from the corresponding analyses in our study. The y-axis labels indicate the mean enrichment fold. The black error bar is the standard error of the enrichment fold. Significance was indicated by *** for *P* < 0.001. **(c)** Results of genomic predictions on the individuals of several pig breeds from PGRP with large phenotype differences for teat number (M_TNUM), which based on a linear mixed model and effect information from suggestive lead variants (*P* < 1×10^-5^) of cross-breed meta-GWAS of TNUM. The x-axis labels indicate the pig breeds. The y-axis labels indicate the genomic estimated breeding values (GEBVs). The black error bar is the standard error of the GEBVs. **(d-e)** The prediction accuracy of average daily gain **(d)** and backfat thickness **(e)** in the independent populations using the lead SNPs identified from within-breed meta-GWAS and cross-breed meta-GWAS. The figures below the bars represent the number of SNPs used. The *P*-value was calculated by two-sided Student t-test, ***P* < 0.01 and ****P* < 0.001. **(f)** Proportion of QTL regions that overlap with QTL reported in the Pig QTLdb [19]. The x-axis represents the different significance threshold that grouped by the -$\log_{10} (P)$. T and F indicate QTL regions overlap at least 1bp with QTL reported in the Pig QTLdb or not, respectively. The figures in x-lab indicated the number of QTL within the current significant threshold interval. **(g)** Distribution of proportion of SNPs with inconsistent direction of effect in the tested populations. The x-axis from left to right represents randomly selected SNPs, SNPs detected only in individual-GWAS (no QTL region overlap with meta-GWAS), SNPs detected in both individual-GWAS and meta-GWAS. Significance of differences between groups in **(a, g)** were estimated using the *t.test* function in R (v4.1.2).


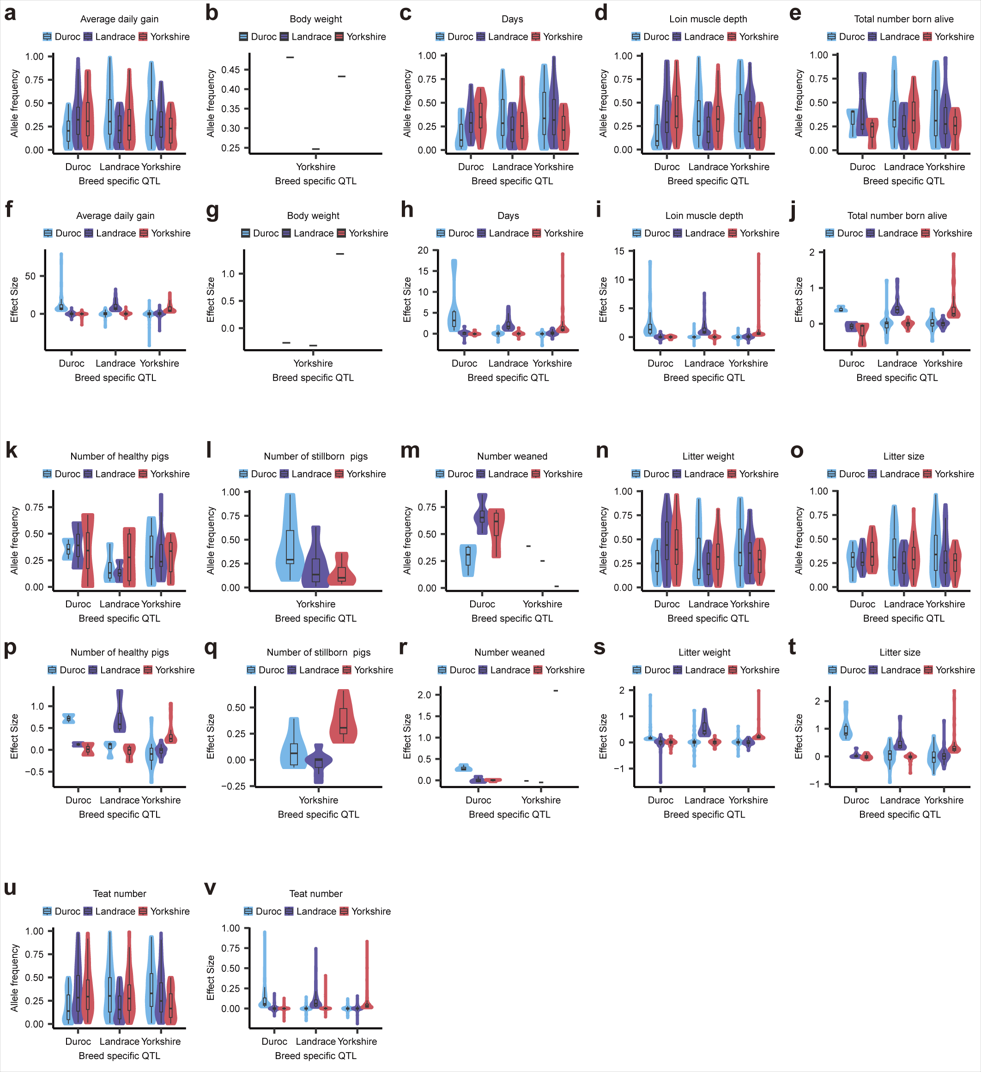


**Figure S5. Comparison of QTL identified from within-breed meta-GWAS across breeds.**

Allele frequency **(a-e, k-o, u)** and effect sizes **(f-j, p-t, v)** distributions of lead SNPs from breed specific QTL across the three breeds for 11 complex traits. Sample sizes for each breed are provided in Table S5. The mean effect sizes of lead SNPs in the observed breed are larger than that from other breeds.


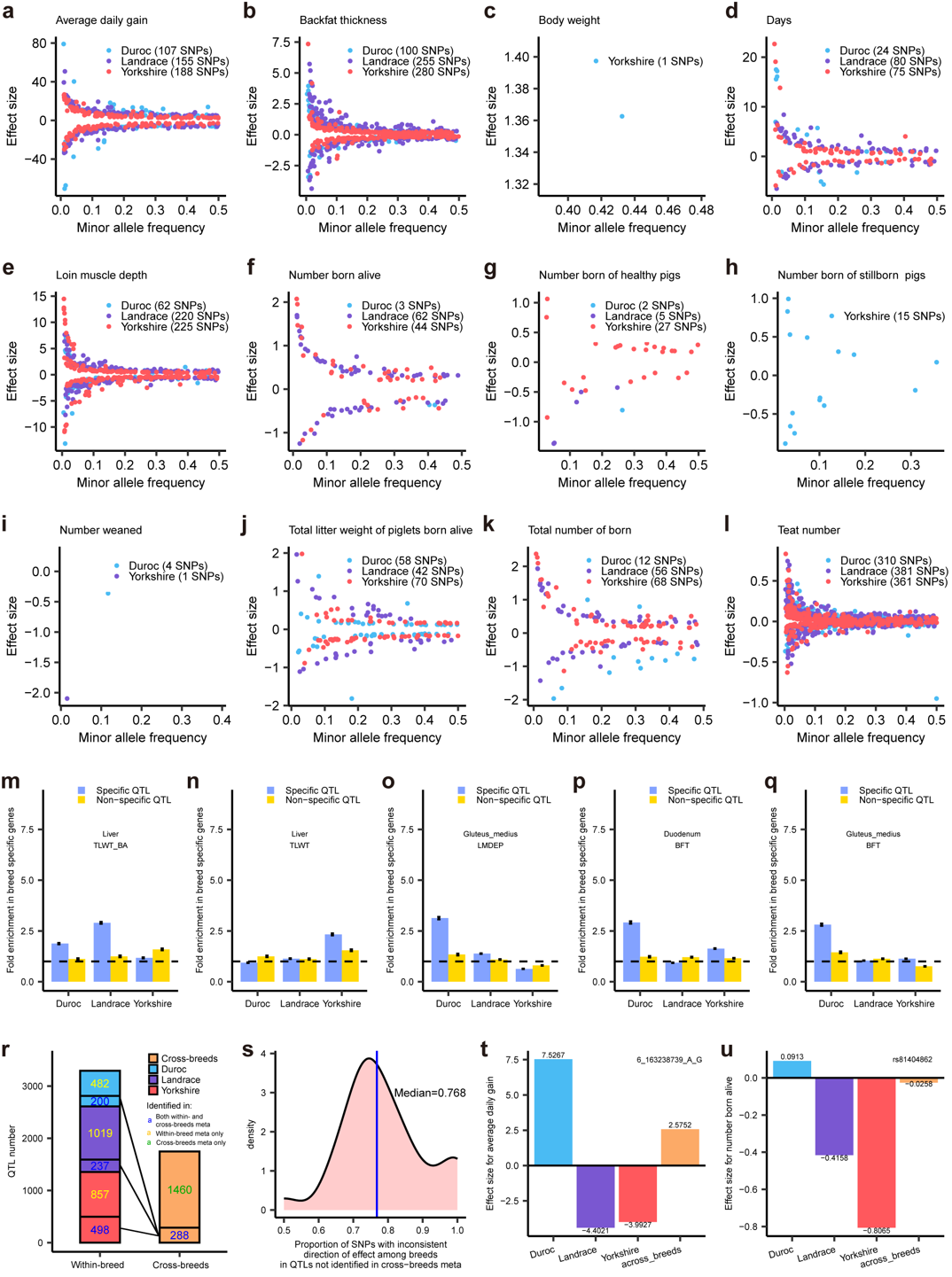


**Figure S6. Cross-breed differences of lead SNPs.**

**(a-l)** Relationship between minor alleles frequency and effect sizes of lead SNPs that were identified in our within-breed GWAS meta-analysis for Duroc, Landrace and Yorkshire pigs. **(m)** Enrichment of breed-specific QTL and non-breed-specific QTL for litter weight in the breed-specific highly expressed genes in liver. The sample sizes of liver for detect breed-specific highly expressed genes in Duroc, Landrace, and Yorkshire were 5, 56, and 62, respectively. **(n-q)** Enrichment of breed-specific QTL and non-breed-specific QTL for complex traits in the breed-specific highly expressed genes in tissue. TLWT: litter weight; LMDEP: loin muscle depth; BFT: backfat thickness. The sample sizes for detect breed-specific highly expressed genes in Duroc, Landrace, and Yorkshire were all 100 in **(n-q)**. The error bar in **(m-q)** represented the standard error of the enrichment fold. **(r)** QTL number for 12 traits in different analyses. X-axis indicates within-breed meta-analysis (including Duroc, Landrace, and Yorkshire) and cross-breed meta-analysis, respectively. The values in different colors indicate the number of QTL. **(s)** Proportion of SNPs with different direction of effect among breeds in QTL regions which detected in within-breed meta-analysis but not detected in cross-breeds meta-analysis among the 12 traits of the three breeds. **(t)** The effect of 6_163238739_A_G on average daily gain in within-breed and cross-breed meta-GWAS. **(u)** The effect of *rs81404862* on number born alive in within-breed and cross-breed meta-GWAS.


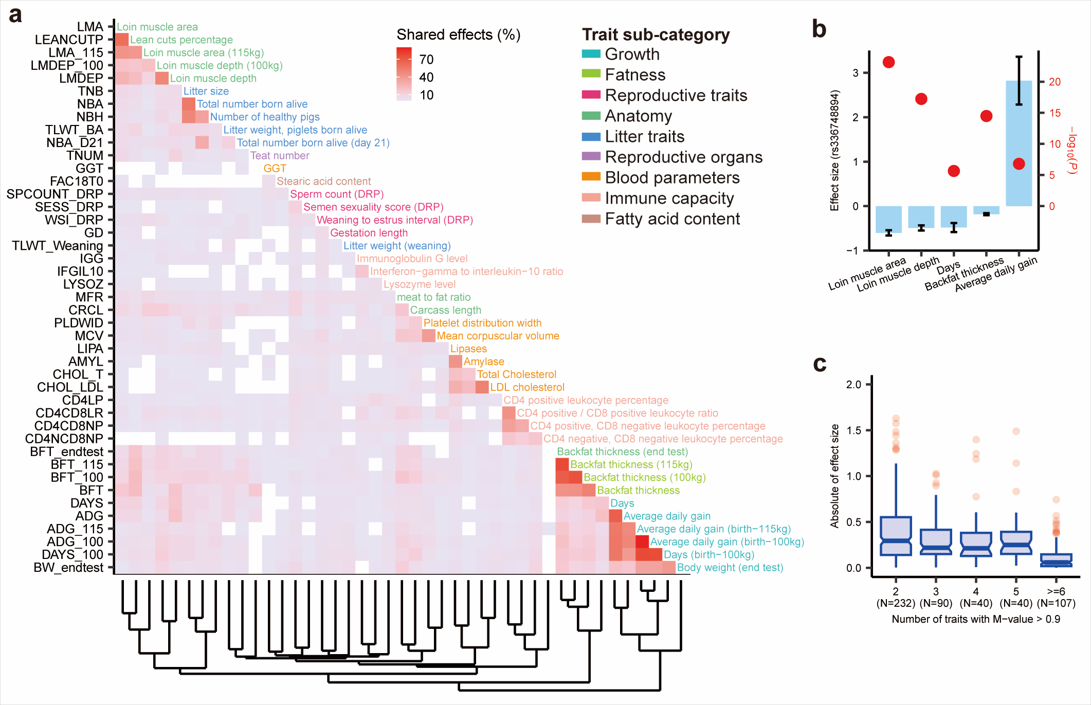


**Figure S7. The pleiotropic of variants across traits.**

**(a)** Clustering for complex traits based on the pleiotropic effects of genetic variants, which was calculated using PLACO [24] based on all lead SNPs from individual-GWAS and meta-GWAS. Clustering was conducted using the *hclust* function in R. **(b)** Effect size and significance of *rs336748894* from cross-breed meta-GWAS on the five complex traits. **(c)** Distribution of the absolute effect size of lead SNPs associated with different numbers of traits (m-value > 0.9) across the cross-breed meta-GWAS of 25 complex traits with large sample size.


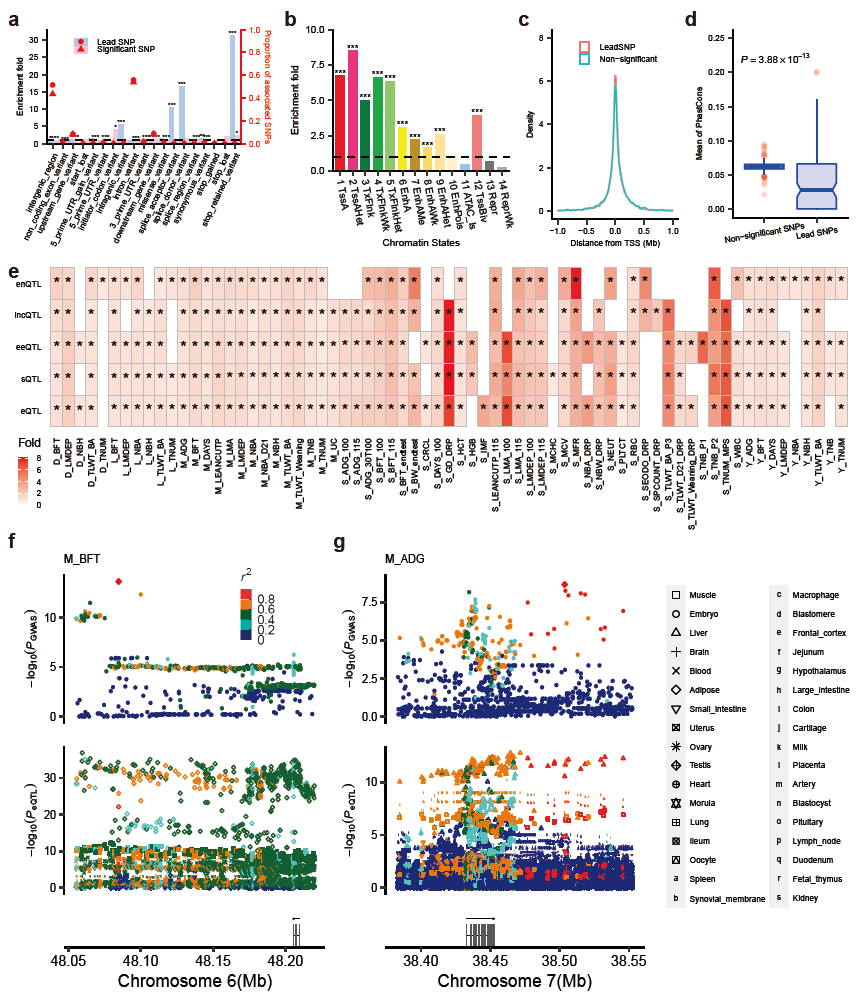


**Figure S8. Annotation and enrichment of lead/significant SNPs (*P* < 5×10^−8^) from within-breed and cross-breed meta-GWAS with sample size > 1,000.**

**(a)** Proportion (red dots, right y-axis) and enrichment fold (bar, left y-axis) of lead SNPs and significant SNPs identified from within-breed and cross-breed meta-GWAS in genomic categories annotated by SNPeff v.4.3 [25]. **(b)** Enrichment of significant SNPs in different categories of regulatory elements, calculated by BEDTools v2.25.0 [28]. **(c)** The distribution of lead SNPs and non-significant SNPs around (± 1Mb) transcript start site (TSS) of protein-coding genes. – and + means upstream and downstream, respectively. **(d)** The mean DNA sequence constraints (PhastCons scores of 100 vertebrate genomes) for lead SNPs and non-significant SNPs across traits. The non-significant SNPs in **(c-d)** were those that matched the MAF (within 0.02) and LD (within 0.1) of the significant SNPs. The *ks.test* function of R (v4.1.2) is used to test the difference between groups. Significance in **(a, b, d)** was indicated by *, ** and *** for *P* < 0.05, 0.01 and 0.001, respectively. **(e)** The heatmap of heritability enrichment for different type of mol-QTL in 69 (which detected significant SNPs) out of 73 meta-GWAS. The “*” indicated that the significant enrichment (*P* < 0.05). The column names indicate trait abbreviations, and the corresponding full names of the traits can be found in Table S5. **(f)** The lead SNP rs1108824455 for backfat thickness (BFT) was also an eQTL regulating *LGALS13* mRNA expression (tissue-specific magnitude = 10) in five tissues (adipose, blood, jejunum, small_intestine, and lung). The top plot shows −log_10_(*P*) of SNPs from GWAS of BFT. The middle plot shows *cis*-eQTL results of *LGALS13* of all 34 tissues. **(g)** The lead SNP rs324200444 for average daily gain (ADG) was also an eQTL modulating *ABCC10* mRNA expression (tissue-sharing magnitude = 33) in four tissues (liver, colon, large_intestine, and muscle). The top plot shows −log_10_(*P*) of SNPs from GWAS of ADG. The middle plot shows *cis*-eQTL results of *ABCC10* for all 34 tissues. Shapes in **(f-g)** indicate different tissues. The filled colors in **(f-g)** represent linkage disequilibrium. The bottom plot in **(f-g)** indicates the positions and strand direction of genes in the locus.


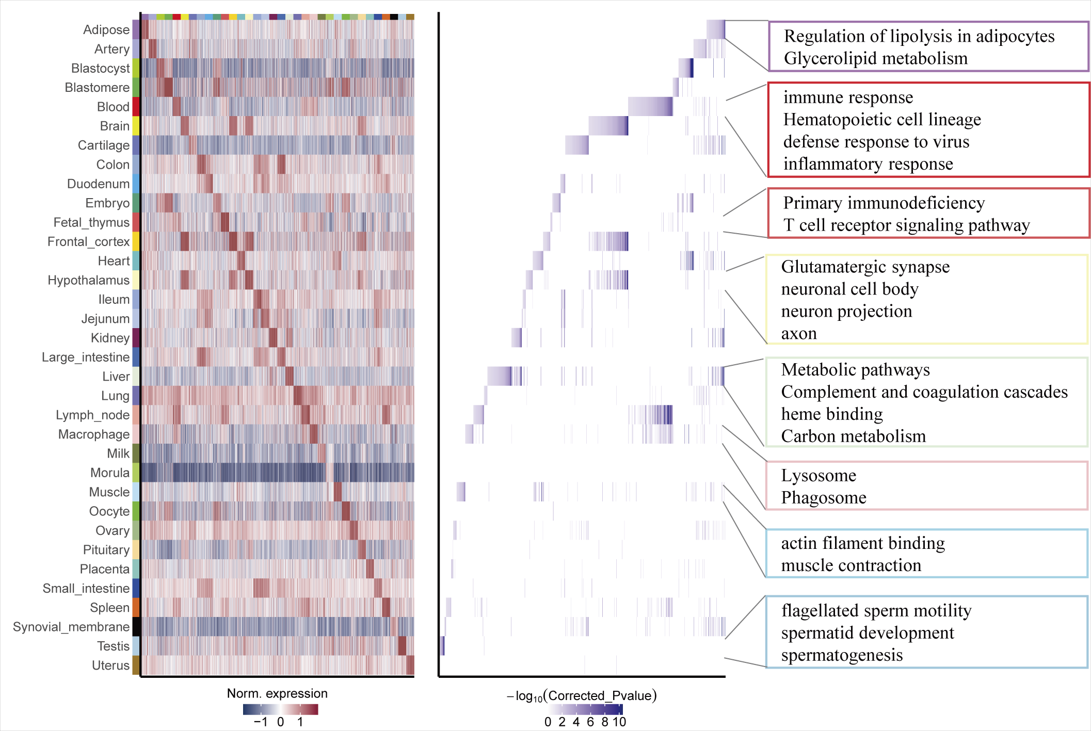


**Figure S9. Tissue-specific highly expressed genes of 34 tissues.**

Expression pattern (left, Standardized Transcripts Per Million, TPM), and enriched Gene Ontology (GO) terms and Kyoto Encyclopedia of Genes and Genomes (KEGG) pathways (right) of the top 1,000 tissue-specific highly expressed genes of 34 tissues.


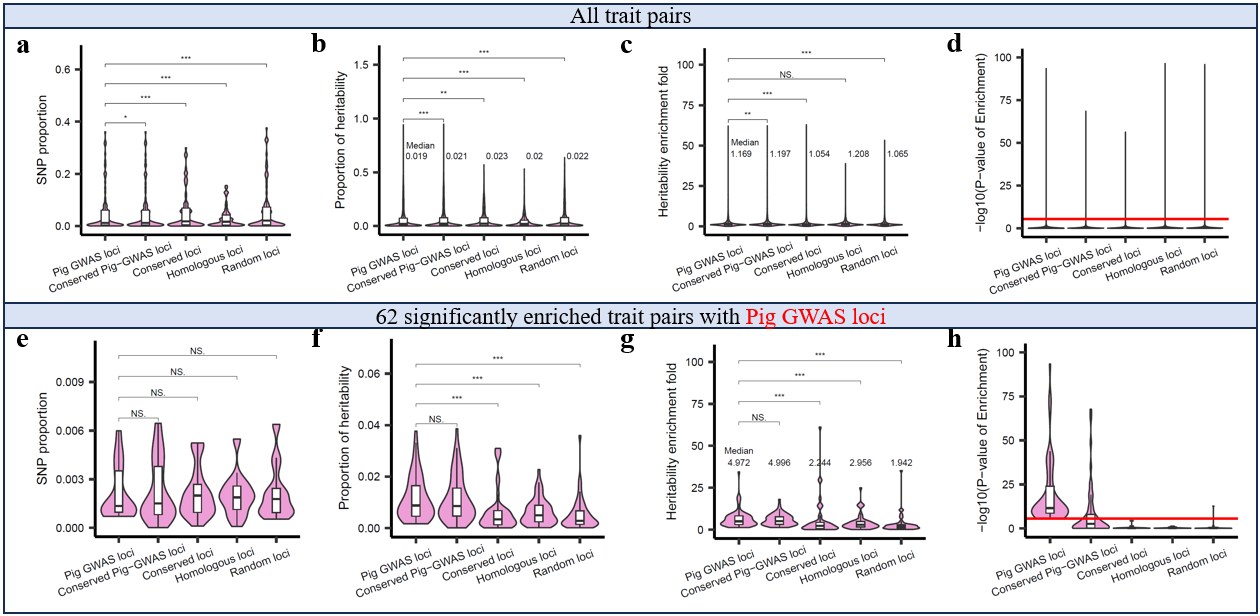


**Figure S10. The heritability enrichment of pig GWAS loci, conserved pig GWAS loci, conserved loci, homologous loci, and random loci in human complex traits. The pig GWAS loci, conserved loci, homologous loci, and random loci were same in the number and length.**

**(a-h)** The distribution of SNPs proportion **(a, e)**, heritability proportion **(b, f)**, heritability enrichment fold **(c, g)**, and -log10 of *P*-value of heritability enrichment **(d, h)** from the heritability enrichment analysis of all human-pig trait pairs **(a-d)** and 62 significantly enriched pig-human trait pairs using pig GWAS loci **(e-h)**. The red line in **(d, h)** represents the significance threshold (*P* = 0.05/126*136 = 2.92 × 10^-6^). The significance is obtained from a two-sided Wilcoxon rank-sum test, **P* < 0.05, ***P* < 0.01 and ****P* < 0.001.

**References**

1. Chang CC, Chow CC, Tellier LC *et al.* Second-generation PLINK: rising to the challenge of larger and richer datasets. *Gigascience* 2015;**4**:7.

2. Teng J, Gao Y, Yin H *et al.* A compendium of genetic regulatory effects across pig tissues. *Nat Genet* 2024;**56**:112–23.

3. Li H, Handsaker B, Wysoker A *et al.* The Sequence Alignment/Map format and SAMtools. *Bioinformatics* 2009;**25**:2078–9.

4. Browning SR, Browning BL. Rapid and Accurate Haplotype Phasing and Missing-Data Inference for Whole-Genome Association Studies By Use of Localized Haplotype Clustering. *Am J Hum Genet* 2007;**81**:1084–97.

5. Browning BL, Zhou Y, Browning SR. A One-Penny Imputed Genome from Next-Generation Reference Panels. *Am J Hum Genet* 2018;**103**:338–48.

6. Bolger AM, Lohse M, Usadel B. Trimmomatic: a flexible trimmer for Illumina sequence data. *Bioinformatics* 2014;**30**:2114–20.

7. Li H, Durbin R. Fast and accurate short read alignment with Burrows–Wheeler transform. *Bioinformatics* 2009;**25**:1754–60.

8. McKenna A, Hanna M, Banks E *et al.* The Genome Analysis Toolkit: A MapReduce framework for analyzing next-generation DNA sequencing data. *Genome Res* 2010;**20**:1297–303.

9. Madsen P, Sørensen P, Su G et al. DMU - a package for analyzing multivariate mixed models. 2006:2014:27-11.

10. Garrick DJ, Taylor JF, Fernando RL. Deregressing estimated breeding values and weighting information for genomic regression analyses. *Genet Sel Evol* 2009;**41**:55.

11. Jiang L, Zheng Z, Fang H *et al.* A generalized linear mixed model association tool for biobank-scale data. *Nat Genet* 2021;**53**:1616–21.

12. Jiang L, Zheng Z, Qi T *et al.* A resource-efficient tool for mixed model association analysis of large-scale data. *Nat Genet* 2019;**51**:1749–55.

13. Winkler TW, Day FR, Croteau-Chonka DC *et al.* Quality control and conduct of genome-wide association meta-analyses. *Nat Protoc* 2014;**9**:1192–212.

14. Bouwman AC, Daetwyler HD, Chamberlain AJ *et al.* Meta-analysis of genome-wide association studies for cattle stature identifies common genes that regulate body size in mammals. *Nat Genet* 2018;**50**:362–7.

15. Willer CJ, Li Y, Abecasis GR. METAL: Fast and efficient meta-analysis of genomewide association scans. *Bioinformatics* 2010;**26**:2190–1.

16. de Leeuw CA, Mooij JM, Heskes T *et al.* MAGMA: Generalized Gene-Set Analysis of GWAS Data. Tang H (ed.). *PLOS Comput Biol* 2015;**11**:e1004219.

17. Cai Z, Christensen OF, Lund MS *et al.* Large-scale association study on daily weight gain in pigs reveals overlap of genetic factors for growth in humans. *BMC Genomics* 2022;**23**:133.

18. Yang J, Lee SH, Goddard ME *et al.* GCTA: A tool for genome-wide complex trait analysis. *Am J Hum Genet* 2011;**88**:76–82.

19. Hu Z-L, Park CA, Reecy JM. Bringing the Animal QTLdb and CorrDB into the future: meeting new challenges and providing updated services. *Nucleic Acids Res* 2022;**50**:D956–61.

20. Wang G, Sarkar A, Carbonetto P *et al.* A Simple New Approach to Variable Selection in Regression, with Application to Genetic Fine Mapping. *J R Stat Soc Ser B Stat Methodol* 2020;**82**:1273–300.

21. Bu D, Luo H, Huo P *et al.* KOBAS-i: intelligent prioritization and exploratory visualization of biological functions for gene enrichment analysis. *Nucleic Acids Res* 2021;**49**:W317–25.

22. Crespo-Piazuelo D, Acloque H, González-Rodríguez O *et al.* Identification of transcriptional regulatory variants in pig duodenum, liver, and muscle tissues. *Gigascience* 2022;**12**, DOI: 10.1093/gigascience/giad042.

23. Han B, Eskin E. Interpreting Meta-Analyses of Genome-Wide Association Studies. Kerr K (ed.). *PLoS Genet* 2012;**8**:e1002555.

24. Ray D, Venkataraghavan S, Zhang W *et al.* Pleiotropy method reveals genetic overlap between orofacial clefts at multiple novel loci from GWAS of multi-ethnic trios. Cordell HJ (ed.). *PLOS Genet* 2021;**17**:e1009584.

25. Cingolani P, Platts A, Wang LL *et al.* A program for annotating and predicting the effects of single nucleotide polymorphisms, SnpEff. *Fly (Austin)* 2012;**6**:80–92.

26. Pan Z, Yao Y, Yin H *et al.* Pig genome functional annotation enhances the biological interpretation of complex traits and human disease. *Nat Commun* 2021;**12**, DOI: 10.1038/s41467-021-26153-7.

27. M N. fmsb: Functions for Medical Statistics Book with some Demographic Data. 2019:2020-03–12.

28. Quinlan AR, Hall IM. BEDTools: a flexible suite of utilities for comparing genomic features. *Bioinformatics* 2010;**26**:841–2.

29. Gel B, Díez-Villanueva A, Serra E *et al.* regioneR: an R/Bioconductor package for the association analysis of genomic regions based on permutation tests. *Bioinformatics* 2016;**32**:289–91.

30. Neph S, Kuehn MS, Reynolds AP *et al.* BEDOPS: high-performance genomic feature operations. *Bioinformatics* 2012;**28**:1919–20.

31. Navarro Gonzalez J, Zweig AS, Speir ML *et al.* The UCSC Genome Browser database: 2021 update. *Nucleic Acids Res* 2021;**49**:D1046–57.

32. Bulik-Sullivan BK, Loh P-R, Finucane HK *et al.* LD Score regression distinguishes confounding from polygenicity in genome-wide association studies. *Nat Genet* 2015;**47**:291–5.

33. Speed D, Holmes J, Balding DJ. Evaluating and improving heritability models using summary statistics. *Nat Genet* 2020;**52**:458–62.

34. Urbut SM, Wang G, Carbonetto P *et al.* Flexible statistical methods for estimating and testing effects in genomic studies with multiple conditions. *Nat Genet* 2019;**51**:187–95.

35. Finucane HK, Reshef YA, Anttila V *et al.* Heritability enrichment of specifically expressed genes identifies disease-relevant tissues and cell types. *Nat Genet* 2018;**50**:621–9.

36. Pividori M, Rajagopal PS, Barbeira A *et al.* PhenomeXcan: Mapping the genome to the phenome through the transcriptome. *Sci Adv* 2020;**6**, DOI: 10.1126/sciadv.aba2083.

37. Barbeira AN, Dickinson SP, Bonazzola R *et al.* Exploring the phenotypic consequences of tissue specific gene expression variation inferred from GWAS summary statistics. *Nat Commun* 2018;**9**:1–20.

38. Barbeira AN, Pividori M, Zheng J *et al.* Integrating predicted transcriptome from multiple tissues improves association detection. Plagnol V (ed.). *PLOS Genet* 2019;**15**:e1007889.

39. Zhu Z, Zhang F, Hu H *et al.* Integration of summary data from GWAS and eQTL studies predicts complex trait gene targets. *Nat Genet* 2016;**48**:481–7.

40. Shirai Y, Nakanishi Y, Suzuki A *et al.* Multi-trait and cross-population genome-wide association studies across autoimmune and allergic diseases identify shared and distinct genetic component. *Ann Rheum Dis* 2022;**81**:1301–12.
