# Supplementary figures and images for: Integrating large-scale meta-GWAS and PigGTEx resources to decipher the genetic basis of 232 complex traits in pigs
Source: Natl Sci Rev. 2025 Feb 17;12(5):nwaf048. doi: 10.1093/nsr/nwaf048 (PMC12051865; doi:10.1093/nsr/nwaf048)

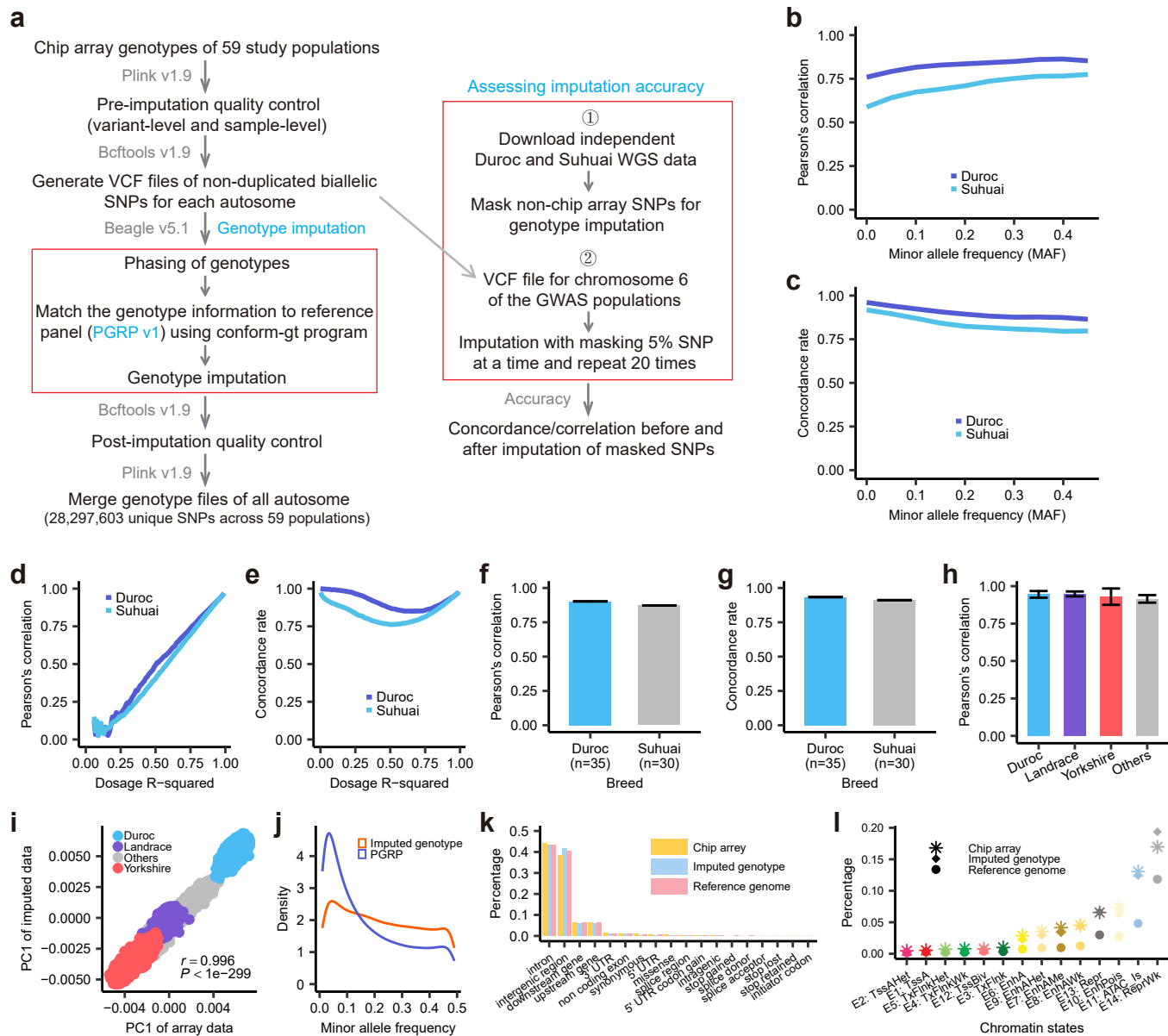

Supplement: nwaf048_Supplemental_Files [file nwaf048_supplemental_files.zip › Fig. S1 genotype imputation.pdf]

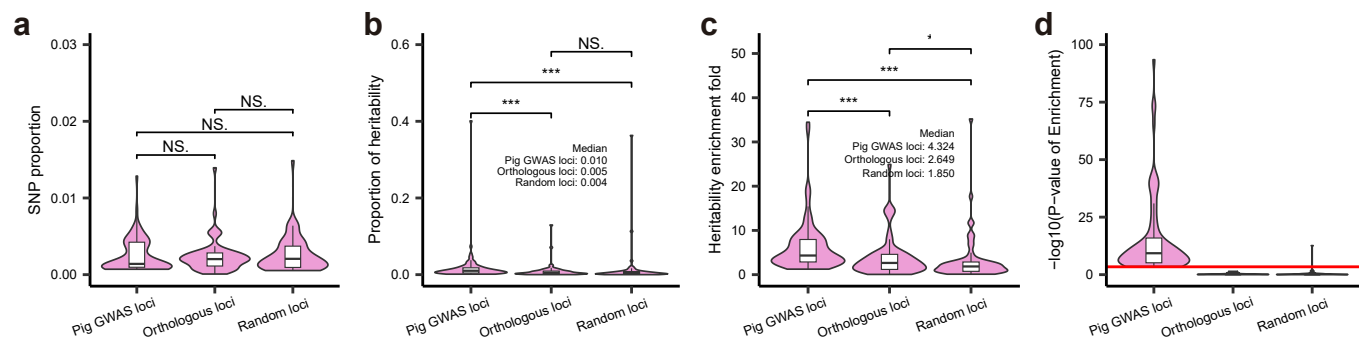

Supplement: nwaf048_Supplemental_Files [file nwaf048_supplemental_files.zip › Fig. S10.pdf]

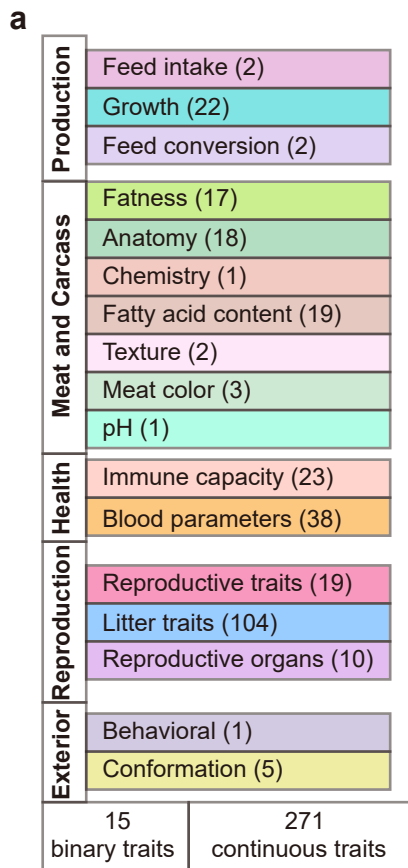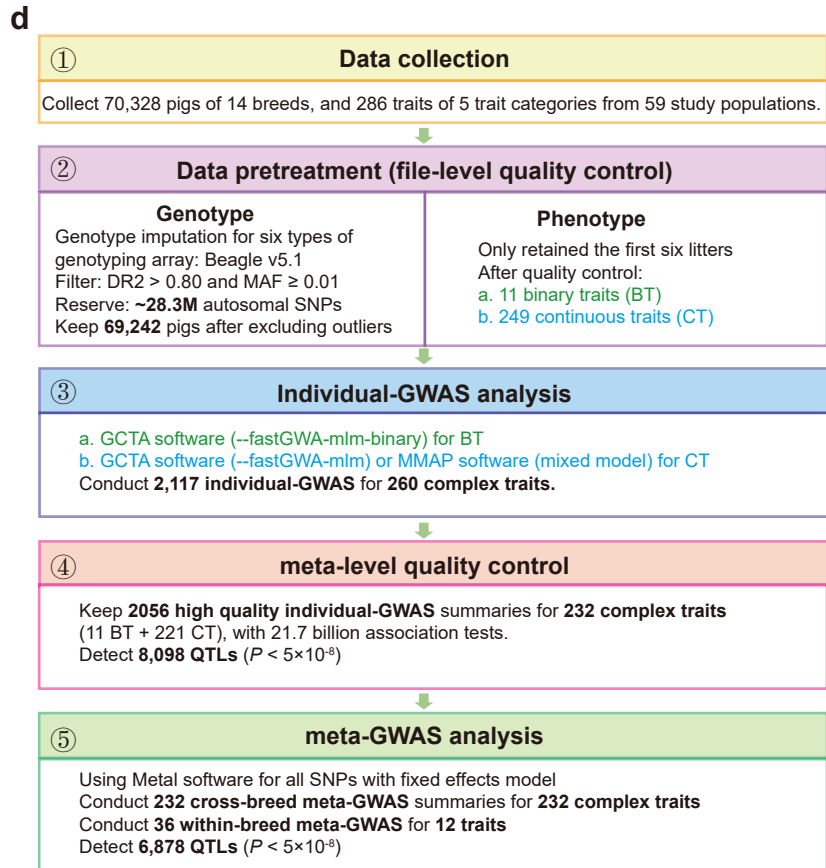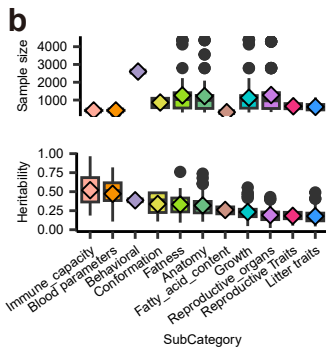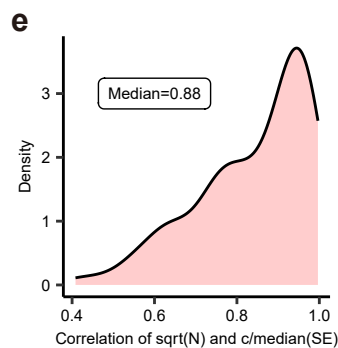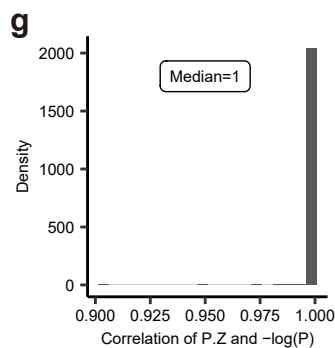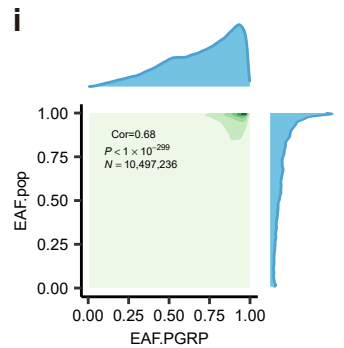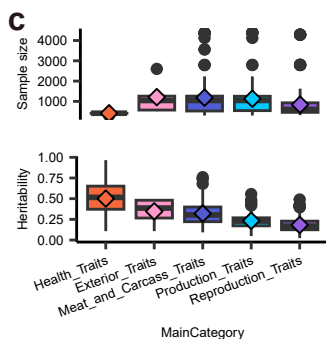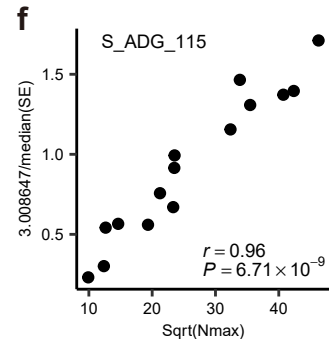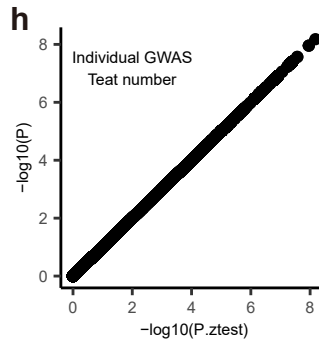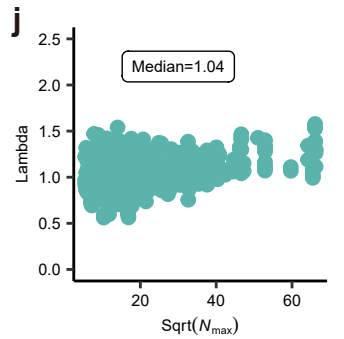

Supplement: nwaf048_Supplemental_Files [file nwaf048_supplemental_files.zip › Fig. S2 individual GWAS.pdf]

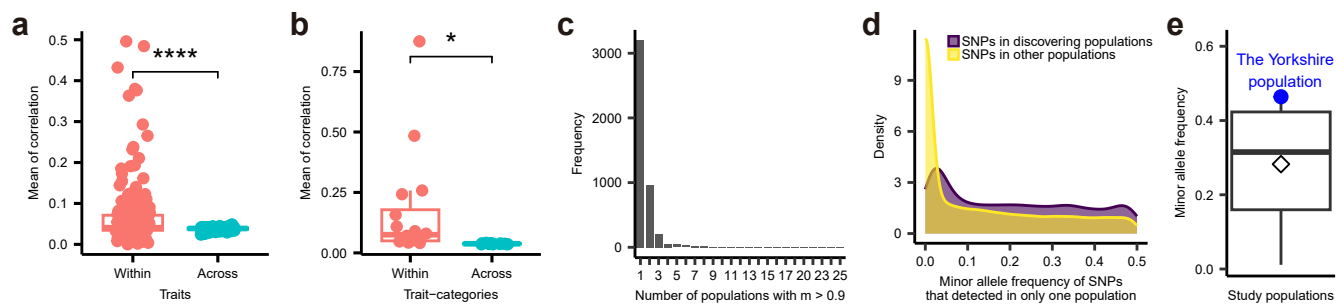

Supplement: nwaf048_Supplemental_Files [file nwaf048_supplemental_files.zip › Fig. S3 Lead SNPs in individual GWAS.pdf]

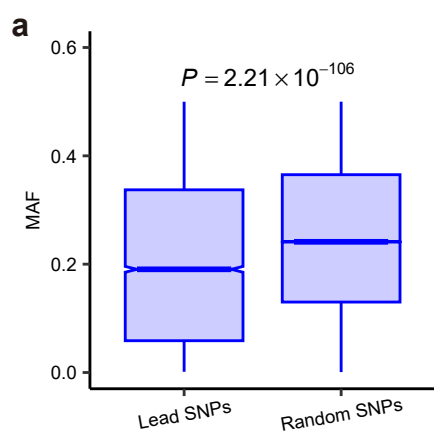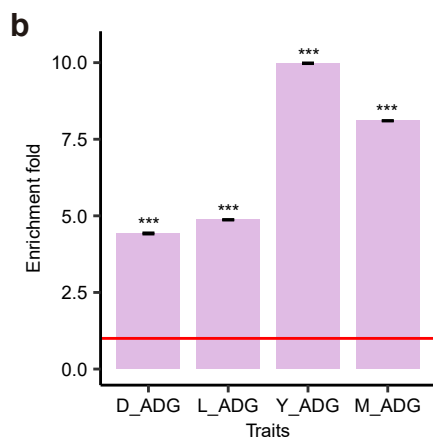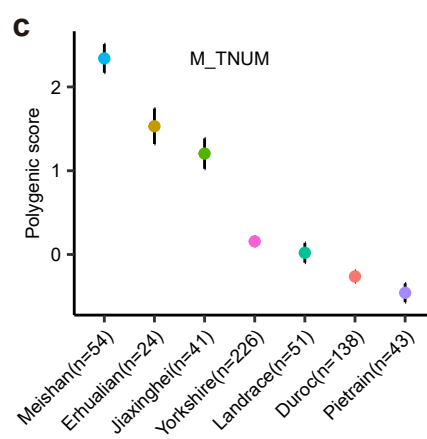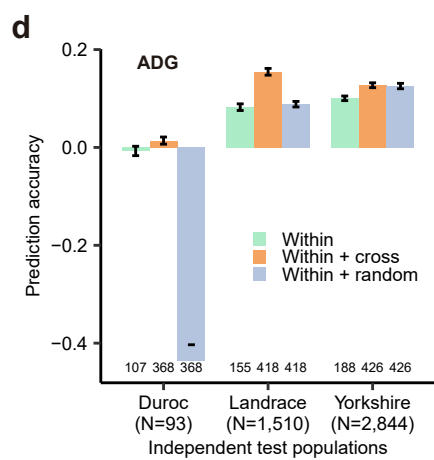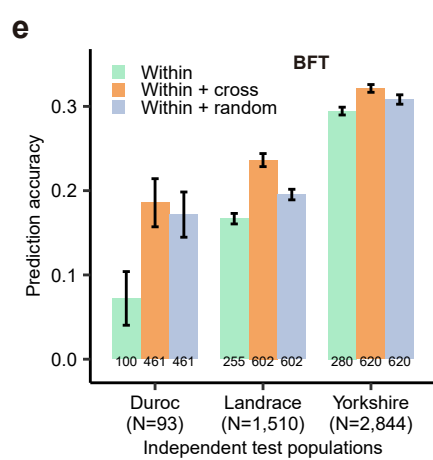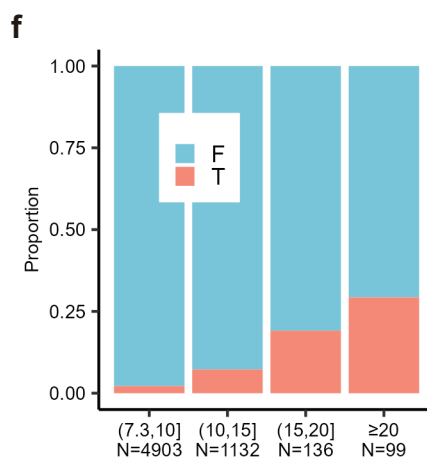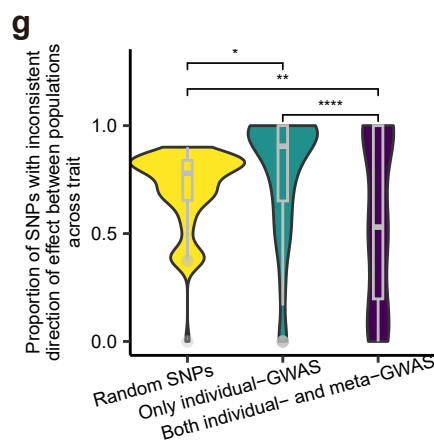

Supplement: nwaf048_Supplemental_Files [file nwaf048_supplemental_files.zip › Fig. S4 Lead SNPs and QTL in meta-GWAS.pdf]

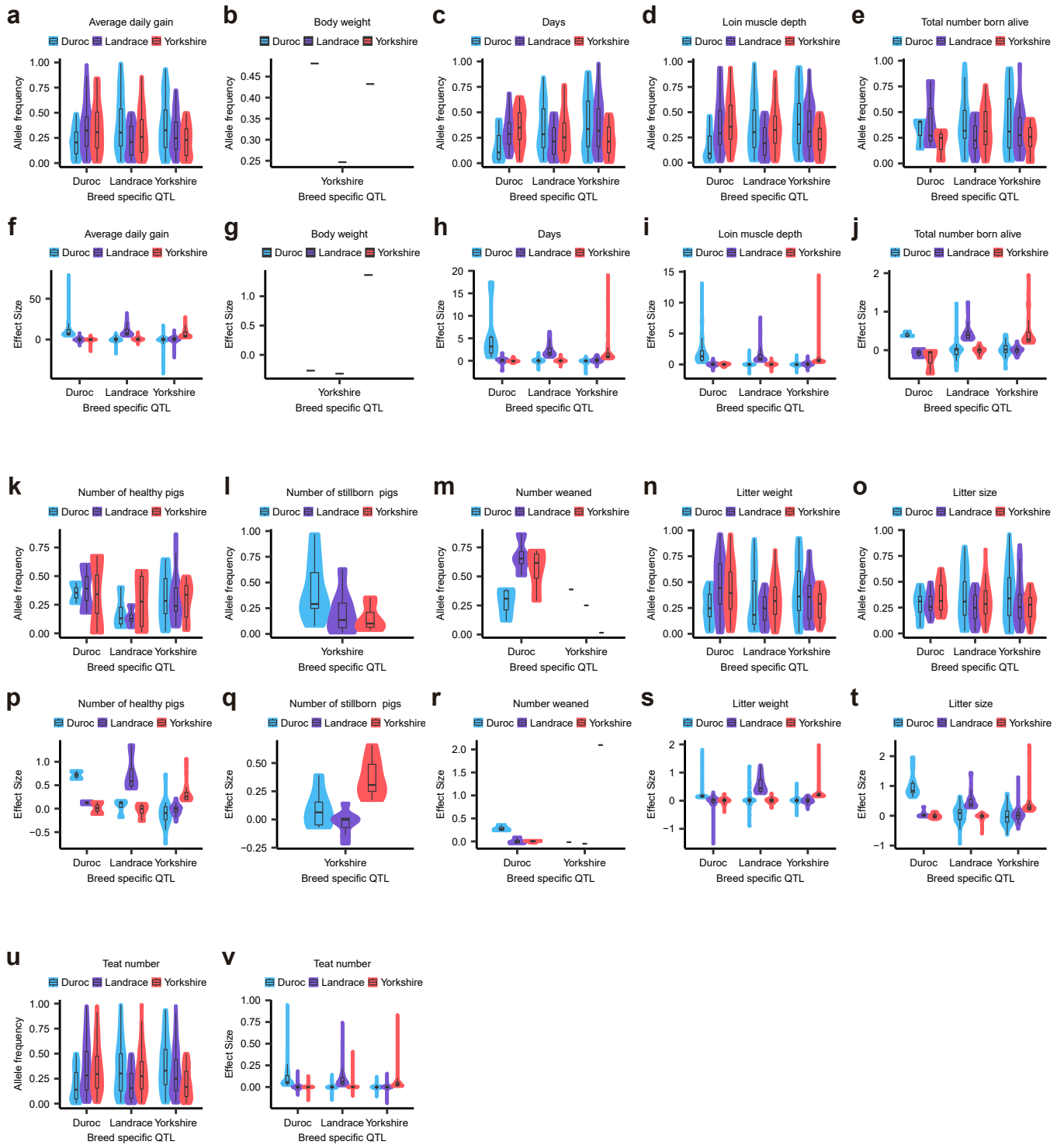

Supplement: nwaf048_Supplemental_Files [file nwaf048_supplemental_files.zip › Fig. S5 Breed QTL AF Effect Size.pdf]

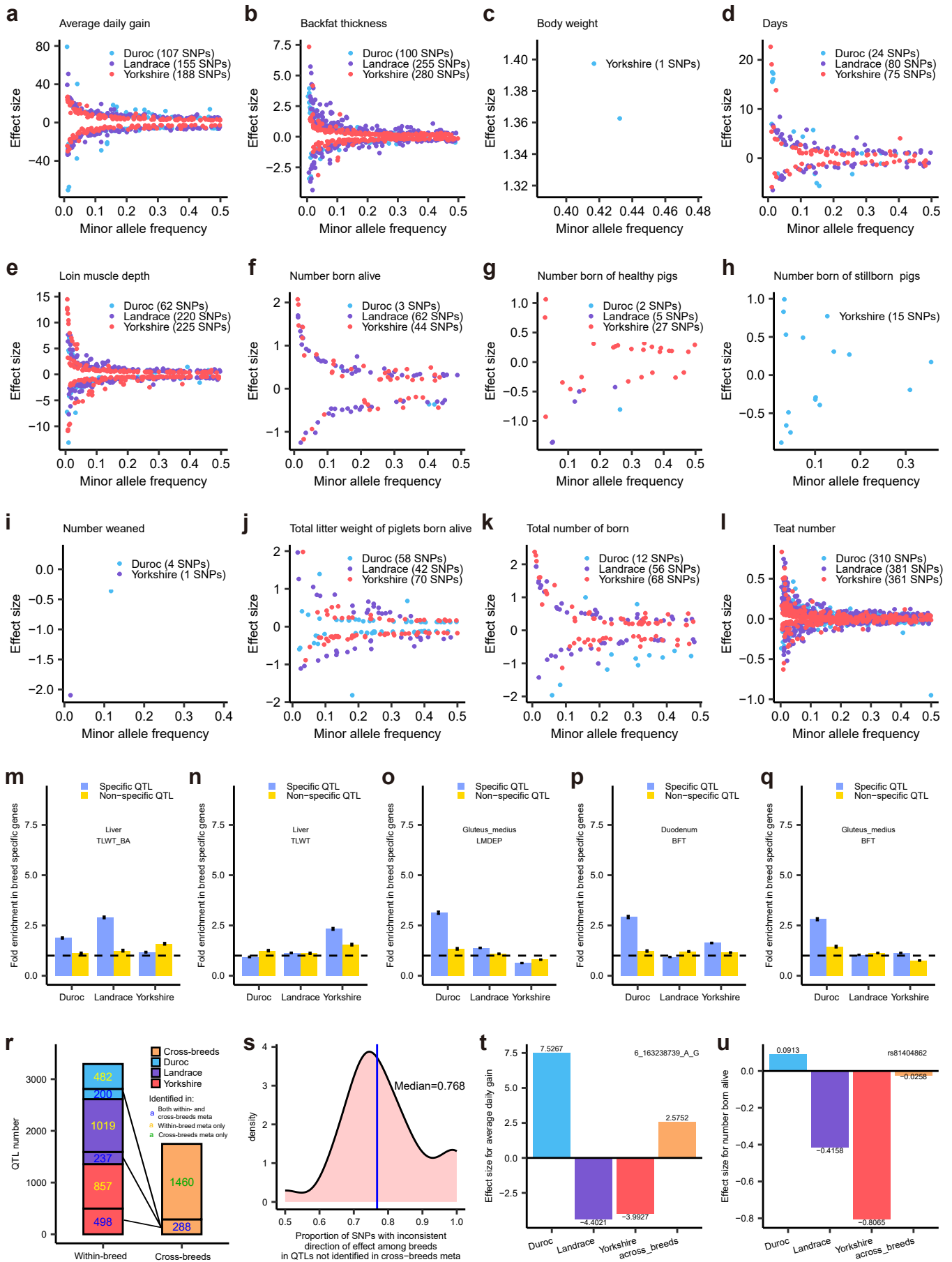

Supplement: nwaf048_Supplemental_Files [file nwaf048_supplemental_files.zip › Fig. S6 Cross-breed Lead SNPs.pdf]

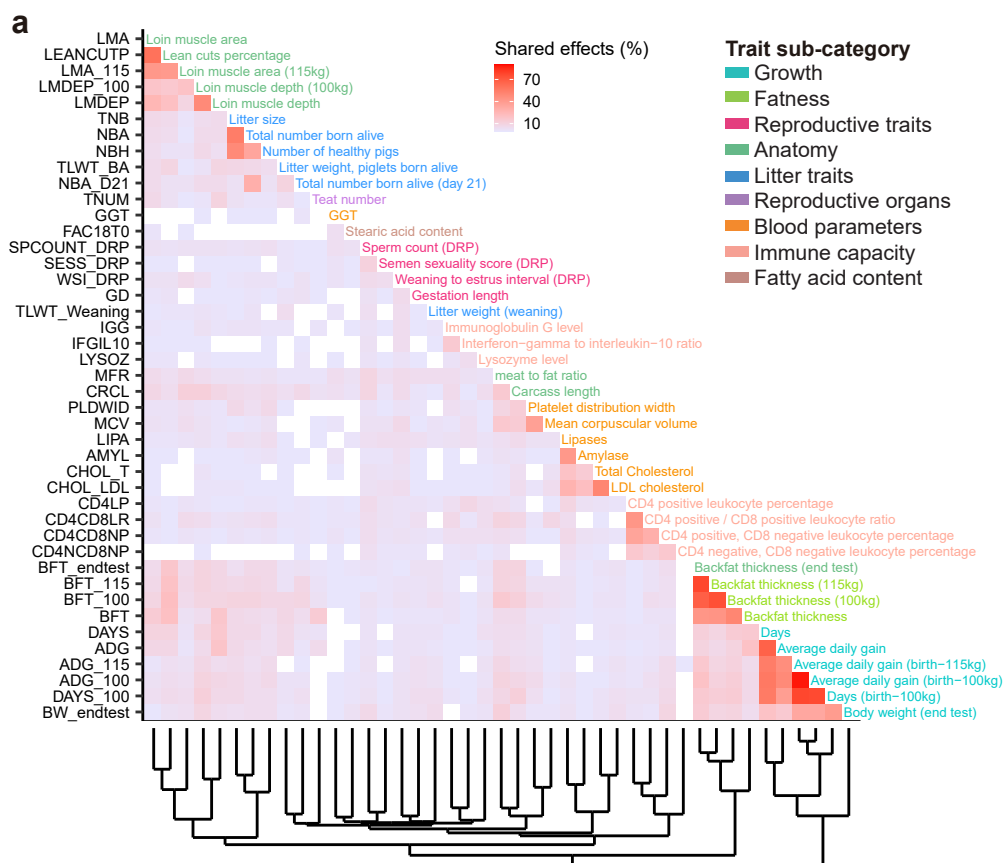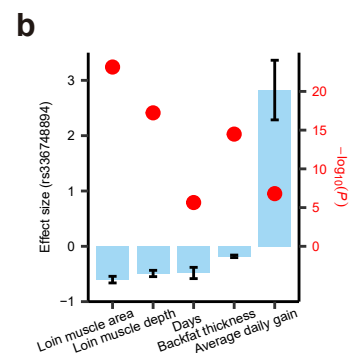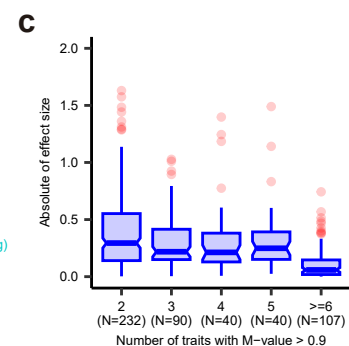

Supplement: nwaf048_Supplemental_Files [file nwaf048_supplemental_files.zip › Fig. S7 pleiotropic of variants across traits.pdf]

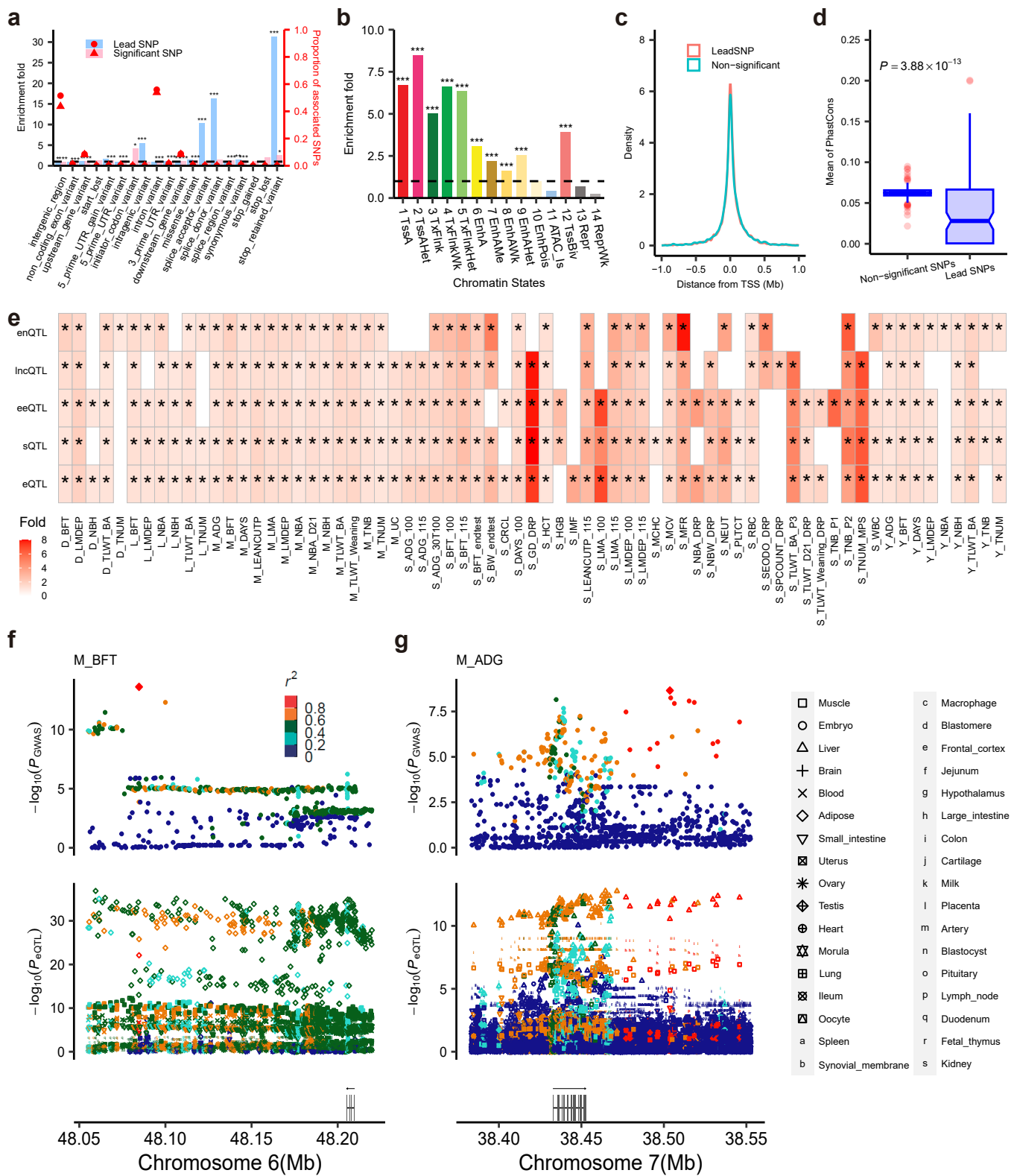

Supplement: nwaf048_Supplemental_Files [file nwaf048_supplemental_files.zip › Fig. S8 Annotation and enrichment of lead SNPs.pdf]

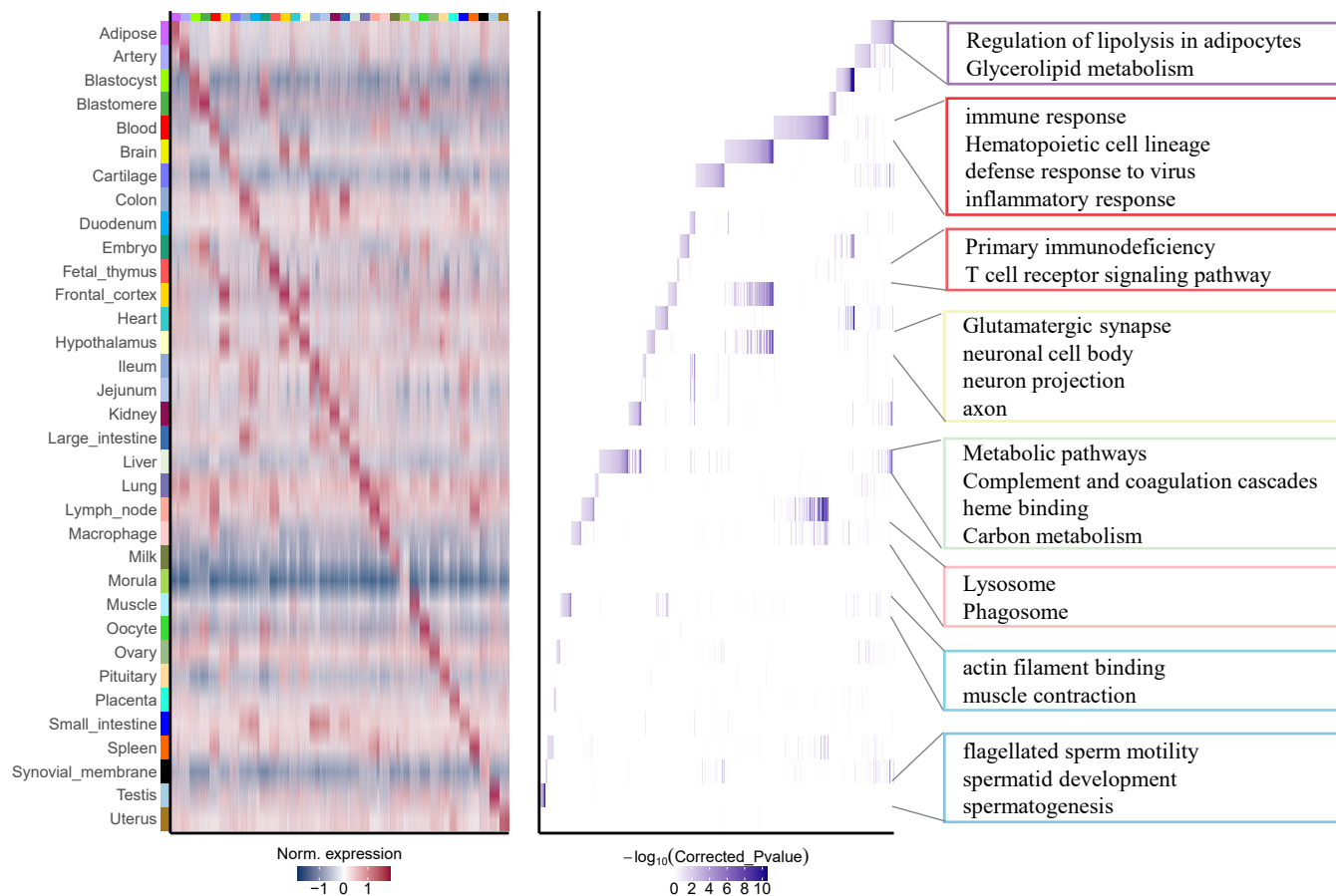

Supplement: nwaf048_Supplemental_Files [file nwaf048_supplemental_files.zip › Fig. S9 Special Gene GO_KEGG KOBAS.pdf]
